# Epigenome-wide association study of plasma lipids in West Africans: the RODAM study

**DOI:** 10.1016/j.ebiom.2023.104469

**Published:** 2023-02-13

**Authors:** Eva L. van der Linden, Karlijn A.C. Meeks, Felix Chilunga, Charles Hayfron-Benjamin, Silver Bahendeka, Kerstin Klipstein-Grobusch, Andrea Venema, Bert-Jan van den Born, Charles Agyemang, Peter Henneman, Adebowale Adeyemo

**Affiliations:** a Department of Public and Occupational Health, Amsterdam UMC, University of Amsterdam, Amsterdam Public Health Research Institute, Amsterdam, the Netherlands; b Department of Vascular Medicine, Amsterdam UMC, University of Amsterdam, Amsterdam Cardiovascular Sciences, Amsterdam, the Netherlands; c Center for Research on Genomics and Global Health, National Human Genome Research Institute, National Institutes of Health, Bethesda, MD, USA; d Department of Physiology, University of Ghana Medical School, Accra, Ghana; e Department of Anesthesia and Critical Care, Korle Bu Teaching Hospital, Accra, Ghana; f MKPGMS-Uganda Martyrs University, Kampala, Uganda; g Julius Global Health, Julius Center for Health Sciences and Primary Care, University Medical Center Utrecht, Utrecht University, the Netherlands; h Division of Epidemiology and Biostatistics, School of Public Health, Faculty of Health Sciences, University of the Witwatersrand, Johannesburg, South Africa; i Department of Human Genetics, Genome Diagnostics Laboratory Amsterdam, Reproduction & Development, Amsterdam UMC, University of Amsterdam, Amsterdam, the Netherlands

**Keywords:** Methylation, Epigenome-wide association study, Cholesterol, Lipids, Sub-Saharan Africa, West Africa, RODAM

## Abstract

**Background:**

DNA-methylation has been associated with plasma lipid concentration in populations of diverse ethnic backgrounds, but epigenome-wide association studies (EWAS) in West-Africans are lacking. The aim of this study was to identify DNA-methylation loci associated with plasma lipids in Ghanaians.

**Methods:**

We conducted an EWAS using Illumina 450k DNA-methylation array profiles of extracted DNA from 663 Ghanaian participants. Differentially methylated positions (DMPs) were examined for association with plasma total cholesterol (TC), LDL-cholesterol, HDL-cholesterol, and triglycerides concentrations using linear regression models adjusted for age, sex, body mass index, diabetes mellitus, and technical covariates. Findings were replicated in independent cohorts of different ethnicities.

**Findings:**

We identified one significantly associated DMP with triglycerides (cg19693031 annotated to *TXNIP*, regression coefficient beta −0.26, false discovery rate adjusted p-value 0.001), which replicated in-silico in South African Batswana, African American, and European populations. From the top five DMPs with the lowest nominal p-values, two additional DMPs for triglycerides (*CPT1A*, *ABCG1*), two DMPs for LDL-cholesterol (*EPSTI1,* cg13781819), and one for TC (*TXNIP*) replicated. With the exception of *EPSTI1*, these loci are involved in lipid transport/metabolism or are known GWAS-associated loci. The top 5 DMPs per lipid trait explained 9.5% in the variance of TC, 8.3% in LDL-cholesterol, 6.1% in HDL-cholesterol, and 11.0% in triglycerides.

**Interpretation:**

The top DMPs identified in this study are in loci that play a role in lipid metabolism across populations, including West-Africans. Future studies including larger sample size, longitudinal study design and translational research is needed to increase our understanding on the epigenetic regulation of lipid metabolism among West-African populations.

**Funding:**

10.13039/501100000780European Commission under the Framework Programme (grant number: 278901).

**Research in context**

**Evidence before this study**

West African populations have a more favourable lipid profile than European populations, with lower levels of plasma triglycerides and higher levels of high-density lipoprotein (HDL) cholesterol, without an accompanying lower prevalence of cardiovascular disease outcomes. These differences might be influenced by DNA methylation. We searched PubMed in January and September of 2021 for articles describing DNA methylation associated with plasma lipids using a combination of Mesh terms “dyslipidaemia”, “cholesterol”, “DNA methylation” and “epigenomics”. Additionally, we searched the EWAS Atlas, a curated database of epigenome-wide association studies, for studies reporting on plasma lipids. We found several publications, including a meta-analysis of cohorts, reporting on methylation loci associated with plasma lipid concentrations. However, few studies were conducted in populations of African ancestry (African Americans), and only one study was conducted in a population-based study in sub-Sahara Africa itself (South African Batswana). None of the studies was conducted in a West African population.

**Added value of this study**

In this epigenome-wide analysis we reported on the association between DNA methylation and plasma lipids conducted in a West African population, i.e. in Ghanaians, a population for whom epigenetic data are scarce. We identified several methylation loci that have previously been linked to lipid metabolism and contribute substantially to the variance in plasma lipid concentration in this Ghanaian population. Several of identified loci replicated in populations of other ethnicities, suggesting that these loci may play a role in lipid metabolism across populations, including West Africans. Additionally, we identified other loci that are potentially relevant in lipid metabolism in Ghanaians specifically. These might contribute to the favourable lipid profile of West African populations, and these may be potentially relevant biomarkers in the pathogenesis of dyslipidaemia.

**Implications of all the available evidence**

The results of this study can serve as a reference for future replication studies and contribute to elucidating mechanisms underlying lipid metabolism in diverse populations. Increasing ethnic diversity in epigenetic research is critical to prevent exacerbation of existing health disparities. Future studies should include larger sample size and a longitudinal study design to increase our pathophysiological understanding of dyslipidaemia among West African populations, thereby informing targeted strategies to curb the rising prevalence of cardiometabolic disorders in sub-Saharan African populations.

## Introduction

Dyslipidaemia is a major risk factor for cardiovascular diseases (CVDs) in general, and ischaemic heart disease in particular.^1^ West African origin populations are considered to have a more favourable lipid profile than other ethnic groups, with lower plasma levels of triglycerides and low-density lipoprotein cholesterol (LDL-C), and higher plasma levels of high-density lipoprotein cholesterol (HDL-C).^2^ However, whereas in high-income western regions mean non-HDL-C levels have decreased over the past few decades, an opposite trend can be observed in most parts of sub-Saharan Africa (SSA),^3^ with a potentially important impact on the CVD burden in this region.

Lipid metabolism is determined by both genetic and environmental factors. Plasma lipid concentrations are 40–60% heritable, but common variants explain only 10–25% of the variance in lipid levels.^4^ Additionally, genome-wide association studies (GWAS) show different loci associated with plasma lipid concentration between African and European origin populations.^5^ Environmental factors such as urbanisation and “westernisation” are shifting patterns in behavioural factors towards less physical activity and more consumption of (fast) food high in salt, sugar, and saturated fat, impacting lipid metabolism.^6^ However, neither genetic variants nor environmental factors alone can completely explain the variation in plasma lipid phenotypes. Gene-environment interaction, mediated by epigenetic modifications, potentially accounts for a proportion of this unexplained variation.^7^ Epigenetic studies facilitate understanding of the regulation of gene expression that occur without changes in the DNA sequence itself.^8^ Several studies have reported on epigenetic processes associated with lipid profiles,^9^ with DNA methylation (DNAm) being studied most widely. While there are few epigenetic studies in African-ancestry populations in general, epigenetics studies in SSA populations are particularly scarce. Only one epigenome-wide association study (EWAS) assessing DNAm in lipid traits has been conducted in an SSA population.^10^ Additionally, as genetic heterogeneity and environmental diversity are large in SSA, epigenetic analyses in other SSA populations can contribute to the discovery of new epigenetic loci associated with lipids. This can improve our understanding of this complex trait in SSA populations, which is highly relevant in the context of CVD prevention. In this study, we aim to identify DNAm loci associated with plasma lipid concentrations in Ghanaians.

## Methods

### Study population and study design

This study used baseline data from the prospective, multicentre Research on Obesity and Diabetes among African Migrants (RODAM) study. Details on this study have been published before^11^ and are summarised here. Between 2012 and 2015, 6385 Ghanaian men and women were recruited in rural Ghana (Ashanti region), urban Ghana (Kumasi and Obuasi), and the European cities of London, Amsterdam, and Berlin. Most participants were of Akan ethnicity, and Ghanaians residing in Europe were first-generation migrants originating from the villages and towns in the Ashanti region. Of those participants aged 25 years and over, with complete data on physical examination and blood sample profile (n = 5659), 736 participants were selected for DNAm profiling (Supplementary Fig. S1). The selection process was based on a case–control design, including about 300 non-drug treated diabetic cases, 300 non-diabetic controls, and 135 non-diabetic, non-obese controls. This sample size was originally chosen to have 80% power to detect a 5% methylation difference between diabetic cases and controls. After exclusion of sex discordances (n = 11), duplicates (n = 8), and those not meeting the quality control thresholds (n = 12), 713 eligible participants remained. Participants with missing data on lipid profile (n = 6), or those using lipid-lowering medication (n = 38) were excluded from the analysis. Additionally, six participants were excluded from the analysis because of outliers in lipid concentrations, resulting in 663 participants included in the current analyses.

### Ethics

Before the start of data collection, ethical approval was obtained from the respective ethics committees of the involved institutions in Ghana (School of Medical Sciences/Komfo Anokye Teaching Hospital Committee on Human Research, Publication & Ethical Review Board, ref. CHRPE/AP/200-12), UK (London School of Hygiene and Tropical Medicine Research Ethics Committee, ref. 6208), the Netherlands (Institutional Review Board of the Academic Medical Center, University of Amsterdam, ref. W12_062#12.17.0086) and Germany (Ethics Committee of Charité-Universitätsmedizin Berlin, ref. EA1/307/12). All participants provided written informed consent before enrolment in the study.

### Phenotypic measurements

Data collection procedures for questionnaire and physical examination were highly standardised across the different study locations, to allow for comparison between the sites. Data on sex, age, and length of stay in Europe were obtained using questionnaires. The use of lipid-lowering medication was based on the Anatomical Therapeutic Chemical classification of medication that participants brought with them to the research location. Physical examination was performed using validated devices. Weight was measured in light clothing without shoes with a SECA 877 scale (Seca GmbH & Co. KG, Hamburg, Germany) to the nearest 0.1 kg. Height was measured without shoes using a SECA 2017 portable stadiometer to the nearest 0.1 cm (Seca GmbH & Co. KG, Hamburg, Germany). Anthropometric measures were taken twice and the mean was used in analyses. Body mass index (BMI) was calculated by dividing the weight in kilograms by the square of the height in meters. Venous blood samples were collected after an overnight fast of at least 10 h. All biochemical analyses were performed in Berlin to avoid inter-laboratory bias. Fasting plasma glucose concentration was measured using the hexokinase method by colorimetry. Diabetes mellitus was defined according to self-reported diabetes and/or fasting glucose ≥7.0 mmol/L. Participants using glucose-lowering medication were excluded from DNAm analysis, because of the potential confounding effect of medication use on methylation profile. A ready-to-use reagent for colorimetry was used to obtain concentrations of total cholesterol (TC), HDL-C, and triglycerides. All analyses were performed using and ABX Pentra 400 chemistry analyser (Horiba ABX SAS, Oberursel, Germany). LDL-C concentration was calculated using the Friedewald equation for individuals with triglyceride levels <4.5 mmol/L. The distribution of the lipid concentration was assessed using histograms and the Shapiro–Wilk test. To ensure normal distribution of the lipid traits, rank-based inverse normal transformation was performed for TC, LDL-C, and HDL-C. Triglyceride concentration was natural log-transformed because of its skewed distribution.

### DNA methylation profiling, processing, and quality control

Source BioScience, Nottingham, UK, conducted the DNA extraction and methylation profiling on participant’s whole blood samples. The process of DNAm profiling, processing, and quality control on RODAM whole blood samples has been described previously.^12^^,^^13^ In short, the Zymo EZ DNAm™ kit (Zymo Research Corp., Irvine, CA, USA) was used for bisulphite conversion of DNA. Using the Infinium® HumanMethylation450 BeadChip (Illumina, San Diego, CA, USA), the converted DNA was amplified and hybridised, thereby quantifying DNAm levels of approximately 485,000 CpG sites. Methylation levels were measured based on the intensities of the methylated and unmethylated probes for each CpG site on the array. These intensities were expressed as methylation Beta-value, which is a value between zero (unmethylated) and one (methylated). A log2 ratio of the intensities of methylated versus unmethylated probes was calculated, which is referred to as M-values. Quality control was performed using the *MethylAid* package (version 1.28.0) in R statistical software (version 4.1.2). The *minfi* package (version 1.40.0) was used for functional normalisation of the raw 450K data. A total set of 429,459 CpG sites remained after removal of probes annotated to the X and Y chromosomes, known to involve cross-hybridisation or to involve common SNPs with a minor allele frequency of ≥5%.^14^ Blood cell mixture estimation was based on the method described by Houseman et al.^15^

### Statistical analyses

#### Association between lipids and DNA methylation

To identify differentially methylated positions (DMPs), the association between lipid concentration (independent variable) and DNAm M-values (dependent variable), were examined using multivariate linear regression analysis using the *lmFit* function of the *Limma* package (version 3.50.1). M-values were used for DMP analyses because of the non-normal distribution of Beta-values. Beta-values were reported for visualisation and to help interpretation of the results.^16^ Because of correlation with DNAm, sex, age, geographical location, estimated cell count (CD8+, CD4+, natural killer cells, B cells, monocytes, and granulocytes), hybridisation batch and array position were included as covariates in the models, based on principal components analysis (Supplementary Fig. S2). Additionally, BMI and diabetes were included in the model, because of an overrepresentation of participants with diabetes and high BMI in the sample. QQ-plots were used to assess model fit (Supplementary Fig. S3). The DMP analysis was run stratified by geographical location, because of the previously observed large difference in plasma lipid profile between the sites (rural and urban Ghana, London, Amsterdam, and Berlin), and to reduce the impact of unobserved confounding factors that differ between the geographical locations. The results for the EWAS per site were then meta-analysed using METAL statistical software (version 2011-03-25). A fixed-effect model, based on effect size and accompanying standard errors was applied. Direction of effect per site was summarised as ‘+’ for positive effect size, or ‘–‘ for negative effect size. Heterogeneity between the sites was considered significant if the p-value for Chi-squared test for heterogeneity was <0.05. To correct for multiple testing, false discovery rate (FDR) adjusted p-values were calculated using the Benjamini-Hochberg method. FDR-adjusted p-values of <0.05 were considered epigenome-wide significant.

To examine the association between DNA methylation and lipid concentration, as well as the explained variance, the raw Beta-values of the top DMPs for each lipid trait were extracted and used as independent variable in models with untransformed lipid concentration as the dependent variable. Methylation Beta-values were used for this analysis to facilitate interpretation as the increase in plasma lipid concentration in mmol/L per percent increase in methylation Beta-value. The models included the same covariates as the DMP analysis. The multiple R squared statistic of the regression models with and without covariates was used to calculate the variance explained by the DMP. As this analysis was run in the total study population, the analysis was additionally adjusted for geographical location.

#### Replication and transferability

To determine whether the top DMPs with the lowest FDR-adjusted p-values in our study replicated in independent cohorts from different ethnic backgrounds, we performed a look-up using summary statistics from EWAS analyses among Batswana in South Africa,^10^ African Americans in the USA, and European ancestry populations in the USA and Europe.^9^ The criteria for replication were a nominal p-value of <0.05 in the replication cohort and a consistent direction of effect. Supplementary Table S1 provides detailed information on the population and design of the replication studies.

We also evaluated whether findings from populations of different ethnic backgrounds, i.e. South African Batswana, African Americans, and Europeans, were transferable to our Ghanaian study population. Cohort-specific thresholds for epigenome-wide significance were used to determine which CpG sites to extract. For the African Americans and European ancestry populations, these were CpG sites with a Bonferroni adjusted p-value <1∗10ˆ–9 in the meta-analysis by Jhun et al.^9^ For the South African Batswana population, this was a nominal p-value of <1∗10ˆ–5 in the study by Cronjé et al.^10^ The association between lipid concentration and these candidate CpGs was assessed in the Ghanaian study population using linear regression models following the same strategy and covariates as in the DMP analysis. Bonferroni adjusted p-values were calculated for each trait and per ethnic group. Results were considered statistically significant if the p-value was <0.05/*n*_CpGs_.

### Sensitivity analysis

#### Location of residence

As previous RODAM results have shown distinct differences in lipid profiles between Ghanaians in Europe (migrants) and their non-migrating counterparts in Ghana (rural and urban),^17^ a sensitivity analysis was performed to evaluate the effect of the location of residence on our findings. The median DNAm Beta-values for each of the top 5 CpGs as identified in the DMP analysis were compared between the geographical locations, using the Kruskal–Wallis test because of the non-normal distribution of Beta-values.

#### Excluding participants with diabetes mellitus

To examine the impact of diabetes status on our findings, we re-fitted the DMP regression model in a subsample of participants without diabetes (n = 432 participants). The summary statistics of the top CpGs from the EWAS in the total population were then compared to the summary statistics in the subsample of participants without diabetes.

### Biological relevance

The function *gaphunter* within the *minfi* package was used to examine whether the top DMPs were potentially under the influence of a genetic variation. The function was run with a threshold of 0.05, reflecting a gap of 5% in Beta-value, suggestive of genetic influence. Identified CpGs with gaps were next searched in the GoDMC database,^18^ to see whether they have previously been correlated to genetic variation. To assess whether genes annotated to our top DMPs have previously been linked to lipid traits, GeneCards,^19^ the GWAS catalog,^20^ and the EWAS atlas^21^ were examined.

To evaluate the levels of gene expression of the top DMPs per lipid trait as identified in our EWAS, the iMETHYL database was consulted.^22^ This database includes whole-DNA-methylation, whole-genome, and whole-transcriptome data for CD4+ T-lymphocytes, monocytes, and neutrophils collected from about 100 subjects. Gene expression is expressed in log-transformed fragments per kilobase of transcripts per million mapped reads (FPKM). A negative value of FPKM suggests low gene expression, whereas a positive value suggests high expression. Additionally, a search in the EWAS toolkit^23^ was performed, to assess DNA methylation level in subcutaneous and visceral adipose tissue, and in liver tissue for our top DMPs. Pathway enrichment analysis was performed using canonical pathway analysis QIAGEN Ingenuity Pathway Analysis application,^24^ including CpGs that were associated with lipid concentration at a significance level of nominal p-value <1∗10ˆ–4. Pathways with a nominal p-value <0.01, as calculated by the right-tailed Fisher’s Exact Test, were considered to be significantly associated.

### Role of funders

The study funders had no role in the study design, data collection, data analysis, data interpretation or writing of the report. The corresponding author had full access to all the data and the final responsibility to submit for publication.

## Results

### Characteristics of the study population

Population characteristics are described in Table 1. Of the 663 Ghanaian participants, most participants lived in urban Ghana, followed by Amsterdam, London, rural Ghana, and Berlin. More than half of the participants were female and the mean age was 50.7 years. BMI was lowest in participants in rural Ghana and highest in Ghanaians living in London. About one-third of the participants had diabetes mellitus. Regardless of the location of residence, only a small proportion of the participants smoked or drank alcohol. Levels of TC and LDL-C were highest in participants residing in urban Ghana and Europe. In contrast, HDL-C levels were lower, and triglyceride levels were higher in those living in rural Ghana than in the other geographical locations. Population characteristics stratified by sex can be found in Supplementary Table S2.

**Table 1:** Population characteristics.

	Total	Rural Ghana	Urban Ghana	Amsterdam	Berlin	London	p-value^a^
n (% of total)	663	101 (15.2)	239 (36.0)	139 (21.0)	75 (11.3)	109 (16.4)	
Sex, male (%)	281 (42.4)	32 (31.7)	71 (29.7)	83 (59.7)	52 (69.3)	43 (39.4)	<0.001
Age (mean (SD))	50.67 (9.96)	56.21 (8.86)	50.57 (9.77)	48.81 (8.00)	46.68 (10.78)	50.84 (10.94)	<0.001
BMI (mean (SD))	26.73 (5.49)	22.81 (4.35)	26.17 (5.69)	28.21 (4.73)	27.30 (4.34)	29.34 (5.41)	<0.001
Diabetes mellitus (%)	231 (34.8)	40 (39.6)	85 (35.6)	45 (32.4)	28 (37.3)	33 (30.3)	0.619
Alcohol intake (units/day) (median [IQR])	0.00 [0.00, 0.07]	0.00 [0.00, 0.07]	0.00 [0.00, 0.03]	0.00 [0.00, 0.13]	0.13 [0.00, 0.71]	0.00 [0.00, 0.00]	<0.001
Smoking (%)							<0.001
No, but I used to smoke	61 (9.5)	11 (11.3)	22 (9.4)	12 (8.9)	11 (14.9)	5 (4.8)	
No, I have never smoked	569 (88.4)	86 (88.7)	211 (90.2)	120 (88.9)	55 (74.3)	97 (93.3)	
Yes	14 (2.2)	0 (0.0)	1 (0.4)	3 (2.2)	8 (10.8)	2 (1.9)	
Length of Stay in Europe (years) (mean (SD))	18.55 (9.70)	NA	NA	19.00 (7.55)	19.05 (10.38)	17.53 (11.62)	0.464
Blood cell distribution (%) (mean (SD))							
CD8^+^ T lymphocytes	0.11 (0.05)	0.12 (0.05)	0.12 (0.04)	0.10 (0.05)	0.10 (0.05)	0.10 (0.04)	<0.001
CD4^+^T	0.18 (0.06)	0.18 (0.06)	0.18 (0.06)	0.19 (0.05)	0.18 (0.06)	0.18 (0.06)	0.822
NK cells	0.11 (0.06)	0.13 (0.06)	0.11 (0.06)	0.09 (0.05)	0.11 (0.05)	0.10 (0.05)	<0.001
B cells	0.11 (0.03)	0.11 (0.04)	0.11 (0.03)	0.10 (0.03)	0.10 (0.03)	0.10 (0.03)	0.003
Monocytes	0.08 (0.02)	0.08 (0.02)	0.08 (0.03)	0.08 (0.02)	0.08 (0.03)	0.08 (0.02)	0.082
Granulocytes	0.45 (0.09)	0.42 (0.10)	0.44 (0.09)	0.48 (0.09)	0.47 (0.09)	0.47 (0.09)	<0.001
Lipid profile (mmol/L) (median [IQR])							
TC	5.18 [4.43, 5.93]	4.57 [3.91, 5.56]	5.43 [4.58, 6.22]	5.10 [4.39, 5.79]	4.99 [4.54, 6.02]	4.98 [4.53, 5.70]	<0.001
LDL-C	3.30 [2.69, 3.94]	2.79 [2.35, 3.65]	3.56 [2.91, 4.16]	3.24 [2.66, 3.92]	3.13 [2.60, 3.83]	3.18 [2.82, 3.90]	<0.001
HDL-C	1.29 [1.10, 1.51]	1.18 [1.01, 1.36]	1.27 [1.08, 1.50]	1.33 [1.10, 1.60]	1.42 [1.22, 1.66]	1.35 [1.15, 1.55]	<0.001
Triglycerides	0.97 [0.72, 1.38]	1.09 [0.81, 1.47]	1.10 [0.83, 1.54]	0.84 [0.62, 1.16]	0.92 [0.68, 1.35]	0.87 [0.61, 1.12]	<0.001

HDL-C, high-density lipoprotein cholesterol; LDL-C, low-density lipoprotein cholesterol; TC, total cholesterol; SD, standard deviation; IQR, interquartile range.

a p-values represent the comparison between the geographical locations, using one-way ANOVA to compare normally distributed continuous variables, Kruskal–Wallis test for non-normally distributed continuous variables, and Chi-square test for categorical variables.

### Association between lipids and DNA methylation

#### Total cholesterol

None of the CpGs associated with TC concentration was epigenome-wide significant at 5% FDR (Supplementary Fig. S4a). The five CpGs with the smallest nominal p-values (all p-value ≤6∗10ˆ–6), were annotated to the *TXNIP*, the *EPSTI1*, the *LHX9* genes, and to two intergenic CpGs cg11066601 and cg03167407 (Table 2). The associations had generally the same direction of effect in all five geographical locations. An increase in DNAm level of *TXNIP*, was associated with a 4.01 mmol/L decrease in TC level. DNAm levels of cg03167407, the *LHX9,* and the *EPSTI1* DMPs were associated with an increase in TC level ranging from 1.32 to 8.33 mmol/L 9.5% of the variance in TC concentration was attributable to the top CpGs (Table 3).

**Table 2:** Top differentially methylated positions associated with lipids.

**TC**	Regression Coeff^a^	Direction of effect^b^	p-value	FDR adj.pval	chr	Pos	Gene symbol^c^	Gene group	Methylation level, % (sd)^d^
cg19693031	−0.0957	−−−−−	8.43E–07	0.3622	chr1	145441552	*TXNIP*	3′UTR	78.53 (6.36)
cg03753191	0.0997	+++++	3.54E–06	0.6184	chr13	43566902	*EPSTI1*	TSS1500	8.65 (3.11)
cg26816907	0.0718	+++++	5.49E–06	0.6184	chr1	197890812	*LHX9*	Body	29.67 (6.25)
cg11066601	−0.2233	−+−−−	6.53E–06	0.6184	chr1	185373486	Intergenic		78.69 (11.31)
cg03167407	0.1782	+++++	9.30E–06	0.6184	chr2	241261657	Intergenic		77.63 (12.99)
**LDL-C**	**Regression Coeff**	**Direction of effect**	**p-value**	**FDR adj.pval**	**chr**	**pos**	**Gene symbol**	**Gene group**	**Methylation level, % (sd)**
cg03753191	0.0976	+++++	4.14E–06	0.9999	chr13	43566902	*EPSTI1*	TSS1500	8.65 (3.11)
cg26816907	0.0679	+++++	1.36E–05	0.9999	chr1	197890812	*LHX9*	Body	29.67 (6.25)
cg13781819	−0.053	−−−−−	3.40E–05	0.9999	chr1	47469065	Intergenic		88.94 (2.14)
cg20294940	−0.049	−−−−−	5.30E–05	0.9999	chr14	105866596	Intergenic		92.38 (1.61)
cg23970275	–0.0674	−−−−−	5.75E–05	0.9999	chr2	208008052	*KLF7*	Body	16.88 (6.27)
**HDL-C**	**Regression Coeff.**	**Direction of effect**	**p-value**	**FDR adj.pval**	**chr**	**pos**	**Gene symbol**	**Gene group**	**Methylation level, % (sd)**
cg05091570	−0.0746	−−−−−	9.09E–07	0.314	chr1	201709336	*NAV1*	Body	2.83 (0.82)
cg07622193	−0.0624	−−−−−	1.68E–06	0.314	chr19	42701920	Intergenic		11.83 (3.33)
cg00091964	−0.0888	−−−−−	2.19E–06	0.314	chr2	80530891	*CTNNA2*	Body	3.91 (1.46)
cg13767294	−0.072	−−−−−	5.23E–06	0.517	chr17	41856619	*DUSP3*	TSS1500	4.47 (1.13)
cg08926253	0.0481	+++++	6.03E–06	0.517	chr11	614761	*IRF7*	Body	56.44 (4.35)
**Triglycerides**	**Regression Coeff.**	**Direction of effect**	**p-value**	**FDR adj.pval**	**chr**	**pos**	**Gene symbol**	**Gene group**	**Methylation level, % (sd)**
cg19693031	−0.2637	−−−−−	1.67E–09	0.001	chr1	145441552	*TXNIP*	3′UTR	78.53 (6.36)
cg17058475	−0.225	−−−−−	2.09E–06	0.448	chr11	68607737	*CPT1A*	5′UTR	13.91 (5.10)
cg06500161	0.1001	+++++	1.17E–05	0.9999	chr21	43656587	*ABCG1*	Body	61.22 (3.95)
cg05697101	−0.3446	−−−−−	2.81E–05	0.9999	chr2	38829104	*HNRPLL*	Body	8.24 (3.75)
cg11066601	−0.4686	−−−−−	3.34E–05	0.9999	chr1	185373486	Intergenic		78.69 (11.31)

HDL-C, high-density lipoprotein cholesterol; LDL-C, low-density lipoprotein cholesterol; TC, total cholesterol; SD, standard deviation; UTR, untranslated region; TSS, transcription start site.

a For M-values, adjusted for covariates age, sex, BMI, diabetes mellitus, estimated cell count, batch and array position.

b Direction of effect in each of the five sites, represented in order Amsterdam-Berlin-London-Rural Ghana-Urban Ghana; negative sign means negative direction of effect, positive sign means positive direction of effect.

c Annotated using UCSC catalogue.

d Methylation level calculated as: methylation Beta-value∗100.

**Table 3:** Association between DNA methylation of top differentially methylated positions (independent variable) and lipid concentration (dependent variable).

**TC**	*Regress. Coeff. (95% CI)*	*p-value*	*Gene symbol*	*Trait variance (%)*
cg19693031	−4.01 (−5.54;–2.48)	7.43E–07	*TXNIP*	3.5
cg03753191	8.34 (4.75; 11.93)	1.08E–06	*EPSTI1*	2.7
cg03167407	1.32 (0.68; 1.96)	5.83E–05	Intergenic	2.2
cg11066601	−1.30 (−2.04;–0.57)	1.84E–04	Intergenic	1.6
cg26816907	2.93 (1.23; 4.63)	3.42E–04	*LHX9*	1.5
**LDL-C**	**Regress. Coeff.**	**p-value**	**Gene symbol**	**Trait variance (%)**
cg03753191	6.96 (3.9; 10.03)	1.36E–06	*EPSTI1*	2.6
cg20294940	−12.12 (−17.83;–6.40)	2.88E–05	Intergenic	2.3
cg26816907	2.70 (1.25; 4.15)	1.23E–04	*LHX9*	1.8
cg23970275	−3.97 (−5.95;–1.99)	1.36E–04	*LKLF7*	2.0
cg13781819	−6.78 (−10.29;–3.27)	4.92E–04	Intergenic	1.9
**HDL -C**	**Regress. Coeff.**	**p-value**	**Gene symbol**	**Trait variance (%)**
cg08926253	1.42 (0.75; 2.1)	3.73E–05	*IRF7*	2.2
cg07622193	−2.53 (−3.83;–1.24)	8.07E–05	Intergenic	0.9
cg05091570	−8.32 (−12.02;–4.61)	2.26E–04	*NAV1*	2.5
cg00091964	−3.75 (−5.87;–1.63)	2.99E–04	*CTNNA2*	1.6
cg13767294	−3.78 (−6.53;–1.03)	9.14E–03	*DUSP3*	1.0
**Triglycerides**	**Regress. Coeff.**	**p-value**	**Gene symbol**	**Trait variance (%)**
cg19693031	−3.06 (−3.91;–2.22)	1.04E–11	*TXNIP*	6.2
cg06500161	3.20 (1.92; 4.48)	1.38E–06	*ABCG1*	3.1
cg17058475	−2.90 (−4.17;–1.62)	1.45E–06	*CPT1A*	2.5
cg11066601	−0.62 (−1.04;–0.21)	1.21E–03	Intergenic	1.1
cg05697101	−2.04 (−3.63;–0.44)	2.35E–03	*HNRPLL*	0.8

Model = [lipid] (untransformed) ∼ Beta-value + sex + age + blood cell estimate + technical variables + BMI + diabetes + site.

TC, total cholesterol; LDL-C, low-density lipoprotein cholesterol; HDL-C, high-density lipoprotein cholesterol.

#### Low-density lipoprotein cholesterol

None of the CpGs associated with LDL-C concentration was epigenome-wide significant at 5% FDR (Supplementary Fig. S4b). The five CpGs with the smallest nominal p-values (all p-value <6∗10ˆ–5) were annotated to the *EPSTI1*, the *LHX9*, and to the *KLF7* genes, and the intergenic CpGs cg13781819 and cg20294940 (Table 2). The association had the same direction of effect in all five geographical locations.

A one percent (1%) increase in DNAm level was associated with around 6.96 mmol/L increase in LDL-C levels for *EPSTI1* and *LHX9*. For the other three CpGs, an increase in DNAm was associated with a 3.97–12.12 mmol/L decrease in LDL-C level. The top 5 CpGs contributed 8.3% to the variance in LDL-C (Table 3).

#### High-density lipoprotein cholesterol

None of the CpGs was significantly associated with HDL-C concentration at <5% FDR (Supplementary Fig. S4c). The five CpGs with the smallest nominal p-values (all p-value ≤6∗10ˆ–6), were annotated to the *NAV1*, the *CTNNA2*, the *CFAP97*, and the *IRF7* genes, and the intergenic CpG cg07622193 (Table 2). The associations had the same direction of effect in all five geographical locations.

An increase in methylation level of the top DMPs was generally associated with a decrease in HDL-C level (Table 3), with a 1% increase in DNAm being associated with a decrease in HDL-C levels up to 8.3 mmol/L. In contrast, cg08926253 showed a positive association between DNAm and HDL-C (regression coefficient beta 1.42). Overall, 6.1% of the variance in HDL-C concentration was attributable to the five CpGs with the smallest nominal p-value.

#### Triglycerides

DNAm levels of cg19693031, were significantly associated with triglyceride concentrations at an epigenome-wide level (Supplementary Fig. S4d). This CpG is located in the 3’ UTR of the *TXNIP* gene. The other four CpGs with the smallest p-values were not epigenome-wide significantly associated, but all had a nominal p-value of <4∗10ˆ–5. These CpGs were annotated the *CPT1A*, the *ABCG1,* and the *HNRPLL* genes, and intergenic CpG cg11066601 (Table 2). The associations had the same direction of effect across all five geographical locations. 1% higher DNAm levels of the *ABCG1* DMP was associated with a 3.20 mmol/L increase in triglyceride levels. The DNAm levels of the other CpGs were associated with lower levels of triglycerides, ranging from −0.62 mmol/L for cg11066601, to −3.06 mmol/L for the *TXNIP* DMP. The combined effect of the top five CpGs explained 11.0% of the variance in triglyceride concentration (Table 3).

### Replication and transferability

For TC, DMPs were only reported in the study by Cronjé et al. and showed a significant association of DNAm of the *TXNIP* gene in South African Batswana (Table 4). LDL-C was significantly associated with DNAm of *EPSTI1* in African American and European populations, as was cg13781819 in African Americans. For HDL-C, none of the five top DMPs could be replicated in the independent cohorts including participants from South Africa Batswana, African Americans or Europeans (Table 4). For triglycerides, *TXNIP* was replicated in all three ethnic groups. Additionally, *CPT1A* and *ABCG1* were replicated in African American and European descent populations.

**Table 4:** Replication of the top differentially methylated positions per lipid trait in South African, African American and European descent populations.

TC	*CpG*	*chr*	*Pos*	*Gene*	South African		African American		European	
					*Regress.Coeff.*	*p-value*	*Regress.Coeff.*	*p-value*	*Regress.Coeff.*	*p-value*
	**cg19693031**	chr1	1.45E+08	*TXNIP*	**−3.49E–04**	**0.013**	NA	NA	NA	NA
	cg03753191	chr13	43566902	*EPSTI1*	−1.45E–04	0.253	NA	NA	NA	NA
	cg26816907	chr1	1.98E+08	*LHX9*	−2.90E–04	0.116	NA	NA	NA	NA
	cg03167407	chr2	2.41E+08	Intergenic	1.37E–04	0.697	NA	NA	NA	NA
	cg11066601	chr1	1.85E+08	Intergenic	NA	NA	NA	NA	NA	NA
**LDL-C**	**CpG**	**chr**	**pos**	**Gene**	**Regress.Coeff.**	**p-value**	**Regress.Coeff.**	**p-value**	**Regress.Coeff.**	**p-value**
	**cg03753191**	chr13	43566902	*EPSTI1*	−1.57E–04	0.304	**5.70E–05**	**0.003**	**1.80E–05**	**0.025**
	cg26816907	chr1	1.98E+08	*LHX9*	−2.32E–04	0.300	1.87E–05	0.355	1.31E–05	0.332
	**cg13781819**	chr1	47469065	Intergenic	−5.65E–05	0.427	**−2.30E–05**	**0.034**	−1.48E–05	0.119
	cg20294940	chr14	1.06E+08	Intergenic	9.48E–06	0.894	−3.39E–06	0.435	−4.47E–06	0.279
	cg23970275	chr2	2.08E+08	*KLF7*	1.00E–04	0.396	−2.59E–05	0.087	−2.13E–05	0.074
**HDL-C**	**CpG**	**chr**	**pos**	**Gene**	**Regress.Coeff.**	**p-value**	**Regress.Coeff.**	**p-value**	**Regress.Coeff.**	**p-value**
	cg05091570	chr1	2.02E+08	*NAV1*	−4.35E–05	0.549	7.92E–04	0.3	−7.90E–05	0.803
	cg07622193	chr19	42701920	Intergenic	−1.38E–04	0.515	−0.002	0.2	−0.001	0.059
	cg00091964	chr2	80530891	*CTNNA2*	−9.74E–05	0.536	−2.91E–04	0.7	7.53E–04	0.086
	cg13767294	chr17	41856619	*DUSP3*	7.68E–05	0.125	0.001	0.1	9.98E–04	0.008
	cg08926253	chr11	614761	*IRF7*	−6.00E–05	0.908	0.003	0.2	3.93E–04	0.819
**Triglycerides**	**CpG**	**chr**	**pos**	**Gene**	**Regress.Coeff.**	**p-value**	**Regress.Coeff.**	**p-value**	**Regress.Coeff.**	**p-value**
	**cg19693031**	chr1	1.45E+08	*TXNIP*	**−0.048**	**3.94E–05**	**−0.03**	**3.19E–24**	**−0.02**	**1.38E–18**
	**cg17058475**	chr11	68607737	*CPT1A*	−1.70E–04	0.979	**−0.01**	**7.19E–06**	**−0.01**	**1.49E–13**
	**cg06500161**	chr21	43656587	*ABCG1*	0.013	0.305	**0.02**	**1.55E–13**	**0.02**	**1.01E–24**
	cg05697101	chr2	38829104	*HNRPLL*	0.002	0.539	−7.96E–04	0.348	4.24E–04	0.195
	cg11066601	chr1	1.85E+08	Intergenic	NA	NA	7.37E–04	0.863	−0.002	0.436

In bold, DMPs replicated in independent cohort at a nominal p-value <0.05. TC, total cholesterol; LDL-C, low-density lipoprotein cholesterol; HDL-C, high-density lipoprotein cholesterol.

The transferability of lipid DMPs identified in previous EWAS to our study population of Ghanaians was generally low (Supplementary Table S3). Overall, transferability was higher for HDL-C than for LDL-C and triglycerides. Transferability from African Americans to Ghanaians was 14% (1/7 CpGs) for HDL-C, 60% (3/5) for LDL-C, and 9% (4/43 CpGs) for triglycerides. Transferability was even lower from Europeans to our Ghanaian sample with 1% (1/69 CpGs) for HDL-C, 0% (0/15) for LDL-C, and 5% (4/86 CpGs) for triglycerides. CpGs reported in South African Batswana did not transfer to our study population.

### Sensitivity analysis

#### Location of residence

**Fig. 1:**
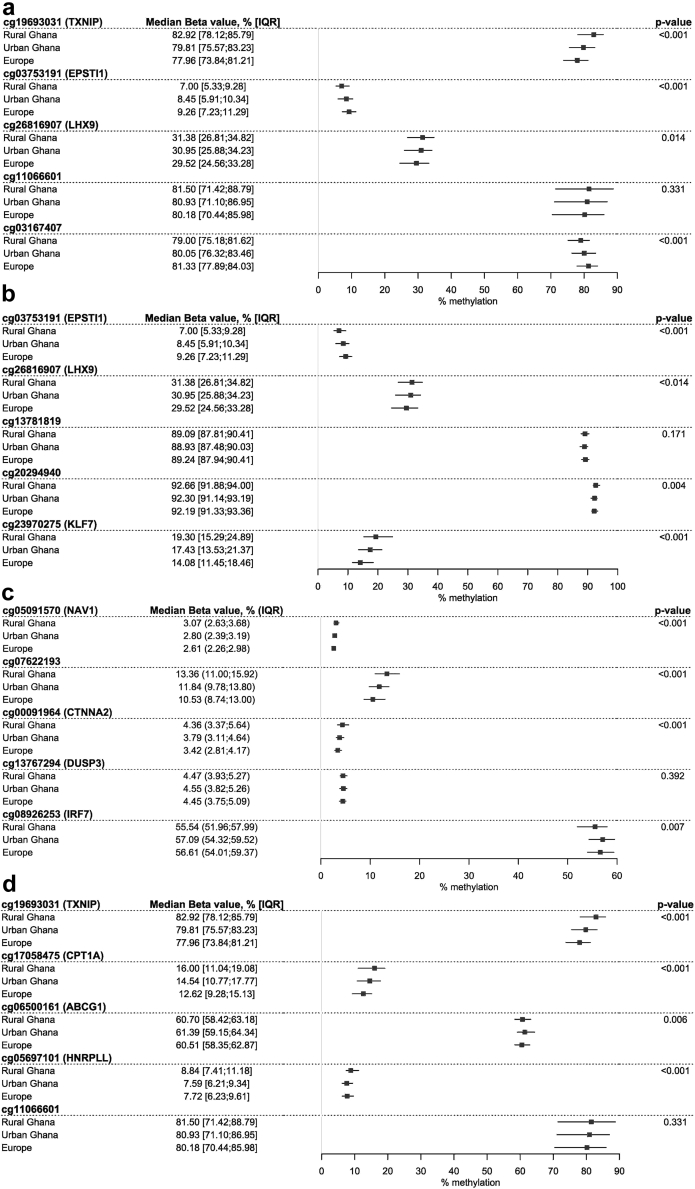
**Methylation levels for top differentially methylated positions stratified associated lipids, stratified by location of residence, for TC (a), LDL-C (b), HDL-C (c), and triglycerides (d)**. Median methylation level with interquartile range (IQR) in percentage, calculated by Beta-value∗100.

For most DMPs, we observed a significant trend in mean methylation level from rural Ghana to urban Ghana to Europe. Across all lipid traits, most of the DMPs were highest methylated in rural Ghana, followed by urban Ghana and Europe, whereas a few showed an opposite trend (Fig. 1a-d). The largest difference was seen for the *TXNIP* and *KLF7* genes, with around 5% lower methylation levels in Europe than in rural Ghana (Fig. 1a and b).

#### Excluding participants with diabetes mellitus

Effect sizes for the top DMPs per lipid trait remained generally the same after excluding participants with diabetes mellitus (Supplementary Table S4).

### Biological relevance

*Gaphunter* identified one DMP with a gap in Beta-value distribution of the intergenic CpG cg03167407 associated with TC concentration. This DMP did not show any association with SNPs in the GoDMC database. The mean methylation levels of the top DMPs for lipid traits from the RODAM study were in line with the methylation levels as reported in the iMETHYL database (Supplementary Table S5). Generally, for those loci that expression data were available for in iMETHYL, low methylation levels of CpGs annotated to the gene body were associated with low gene expression, whereas high methylation in the gene body was associated with high gene expression. The DNA methylation levels in blood, however, did differ from levels reported in subcutaneous and visceral adipose tissue, and in liver tissue as reported in the EWAS toolkit. The pathway enrichment analysis for TC showed enrichment for glutamine biosynthesis, Rho GDP dissociation inhibitor, and actin cytoskeleton signalling pathways. For LDL-C, pathways involved in calcium pathway signalling, and nitric oxide synthase signalling were enriched. Pathway enrichment analysis of HDL-C showed enrichment for the nicotinamide adenine dinucleotide (NAD) biosynthesis pathway. For triglycerides, pathways involved in the lipopolysaccharide/interleukine-1 (LPD/IL-1) inhibition of the retinoid X receptor (RXR), RXR activation, mitochondrial l-carnitine shuttle, and pyroptosis signalling were enriched.

## Discussion

In this EWAS on lipid components in West African populations, we identified one epigenome-wide significant DMP associated with triglycerides (cg19693031 annotated to the *TXNIP* gene). We found that DNAm levels of DMPs annotated to the*TXNIP, NAV1, CPT1A,* and *ABCG1* genes did contribute substantially to the variance in plasma TC, LDL-C, HDL-C, triglyceride concentrations. We were able to replicate our findings in independent cohorts of South African Batswana, African American, and European descent. Additionally, candidate DMPs identified in African American and European populations were transferable to Ghanaians in our study, but not from South African Batswana to Ghanaians. Mean DNAm levels for the top DMPs were generally lower in Ghanaians residing in Europe than in urban or rural Ghana.

Our findings suggest that *TXNIP* methylation is associated with plasma lipids across populations,^9^^,^^10^ including West Africans who have a generally more favourable lipid profile than other populations. DMP cg19693031, located in the 3′UTR of the *TXNIP* gene, was epigenome-wide significantly associated with fasting triglyceride concentration in Ghanaians, and explained 6.2% of the variation in triglyceride levels. In African American and European ancestry populations, this DMP has previously been linked to triglyceride and lipid metabolism,^25^^,^^26^ as well as to other cardiometabolic traits such as weight,^27^ blood glucose,^13^ blood pressure,^28^ and to BMI in a previous RODAM EWAS study.^12^ The *TXNIP* gene encodes for the thioredoxin interacting protein, which is primarily involved in inflammatory, metabolic and apoptotic processes,^29^ and plays an important role in the development of diabetes, by influencing insulin production and beta-cell apoptosis.^30^ The role of TXNIP in lipid metabolism was clearly demonstrated in mouse models, in which TXNIP deficient mice have increased levels of plasma lipids and triglycerides.^31^ Additionally, TXNIP inhibition is a potential target in the treatment of metabolic disorders,^29^ which might be interesting in light of epigenetic regulation of the *TXNIP* gene.

For the TC, LDL-C, and HDL-C, we did not find epigenome-wide significant DMPs. However, we do believe that the top DMPs are potentially relevant associations, as they have previously been described in the regulation of lipids, weight, and glucose metabolism. For instance, for HDL-C, DNAm of cg00091964 annotated to the *CTNNA2* gene has been reported to be associated with TC and LDL-C^26^ and genetic variation in the *CTNNA2* gene has been associated with HDL-C,^32^ BMI^33^, ^34^, ^35^, ^36^ and coronary heart disease^37^ in multi-ethnic populations. In line with our findings DMP cg17058475 (*CPT1A*) and cg06500161 (*ABCG1*) have been linked to triglycerides and to lipid profile in general, BMI, and blood pressure.^25^^,^^26^^,^^28^ For LDL-C, the *KLF7* gene has been linked to BMI,^33^^,^^38^^,^^39^ inflammation,^40^ and subcutaneous adipose tissue^41^ in European origin populations. Additionally, the pathway enrichment analysis showed that our top DMPs were involved in pathways of energy and lipid metabolism, transport and biosynthesis of lipids and cholesterol, and nitric oxide synthase signalling.^42^, ^43^, ^44^, ^45^, ^46^, ^47^, ^48^ Furthermore, the direction of effect and the strength of the associations were similar across all five geographical locations. This shows that despite different contextual factors, similar DMPs are at play in lipid metabolism. Moreover, lipids (independent variable) were not only associated with DNAm (dependent variable), but the methylation levels of the top DMPs (independent variable) were also significantly associated with lipid concentrations (dependent variable). Therefore, to confirm our findings, future research should aim for a larger sample size allowing more statistical power to detect epigenome-wide significant effects.

We were able to replicate findings from Ghanaians in independent cohorts including South African Batswana, African American, and European descent populations, which supports that these DMPs (*TXNIP, CPT1A, ABCG1, EPSTI1)* are potentially relevant in the pathogenesis of dyslipidaemia and are universal across different ancestral groups. In contrast, the transferability of DMPs associated with lipid traits in South African Batswana, African American, and European origin populations to our Ghanaian study population was generally low, but especially limited for the findings in the South African populations. This implies the possible population specificity of these results, which are either based on genetic or environmental differences. Because of the large genetic diversity in SSA, it can be assumed that South African Batswana men are genetically different from Ghanaian population in genes regulating lipids or epigenetics,^49^ thereby making findings less generalisable between different ethnic populations in SSA. In contrast, admixed African Americans have up to 75% shared ancestry with West Africans,^50^ and show a large percentage of European ancestry,^51^ thereby increasing the transferability of findings from African American to Ghanaians. Likewise, Ghanaian migrants residing in Europe share a more similar environment with African Americans and Europeans, whereas environmental factors differ between South African Batswana in South Africa, and Ghanaian migrants in Europe and non-migrants in Ghana, thereby affecting the transferability of findings between ethnicities.

Previous findings from the RODAM study showed lower levels of HDL-C and higher levels of triglycerides in participants residing in rural Ghana, compared to those living in the other locations, and these differences were independent of common risk factors for dyslipidaemia.^17^ In this light, our finding of differences in methylation levels of CpGs between participants living in different geographical locations is interesting. Although we were not able to establish whether these differences in methylation levels are biologically relevant, it does highlight the importance of studying gene-environment interaction in different settings as DNAm is highly dynamic and potentially context-specific.

Dyslipidaemia is strongly related to obesity and diabetes.^52^ This interrelatedness is also demonstrated by the observation that DMPs associated with triglycerides have previously been reported in EWAS on diabetes and obesity in the same Ghanaian study population.^12^^,^^13^ To rule out the potential interacting effects of obesity and diabetes, we adjusted our regression models for these factors. Additionally, in sensitivity analysis, we excluded participants with diabetes, which did not impact the effect size or direction of effect of the association. This indicates that the reported DMPs are potentially involved in lipid metabolism, independent of obesity and diabetes.

The findings of this EWAS study on lipids in a West African population add to our knowledge of epigenetic associations with lipids in diverse populations. Highly standardised data collection across all five geographical locations allowed us to compare DNAm profiles in migrant and non-migrant Ghanaians, thereby assessing the impact of migration on DNAm. Additionally, we were able to perform the EWAS separately per geographical location before meta-analysing the findings, thereby minimising the confounding effect of unknown contextual factors on our results. Even though this study included the largest sample size of a West African population to date, our statistical power to detect epigenome-wide significant DMPs is assumed to be limited. Future studies should aim for a larger sample size, and more EWAS in different SSA populations can contribute to replication and pooling of the results. Because genotyping data were not available, we were not able to adjust our analysis for ancestry principal components. However, as 90% of our study population was of a single ethnolinguistic group (Akan) who have been shown to be genetically homogenous,^53^ it is unlikely that our findings have been significantly affected by population stratification. We assessed DNAm extracted from whole blood samples. Even though lipids are a blood-based trait, methylation patterns can differ in target tissue where metabolism occurs, e.g. in adipocytes or hepatocytes, as our results from the EWAS Toolkit analyses showed. We conducted a cross–sectional association study, and conclusions related to the causal relation between DNAm and plasma lipid concentration should therefore be drawn with caution. For instance, Mendelian randomisation studies have shown lipid levels to be influenced by DNAm,^54^ but also that DNAm can influenced lipid levels.^9^ A longitudinal study design could help to establish temporality and direction of effect.

In conclusion, we identified one epigenome-wide significant DMP associated with triglycerides (*TXNIP*) and several other lipid-associated DMPs (*CPT1A, ABCG1*) in this cohort of Ghanaians, loci which are also associated with lipids in populations of different ancestry. Several other identified CpGs are potentially relevant in lipid metabolism in Ghanaians but further work needs to be done to investigate their association with the observed favourable lipid profile of West African populations. Future studies including larger sample size, longitudinal study design, as well as translational studies - including different tissues and gene expression - can enlarge our pathophysiological understanding of dyslipidaemia among West African populations, thereby informing targeted strategies to curb the rising prevalence of dyslipidaemia in SSA populations.

## Contributors

E.L.L., K.A.C.M., B.J.B., C.A., P.H. and A.A. conceived the study. C.A. and K.A.C.M. designed and carried out the recruitment and data collection. E.L.L., K.A.C.M., and A.A. were responsible for data analysis and interpretation. E.L.L., K.A.C.M. and A.V. verified the underlying data. E.L.L. wrote the article, supervised by K.A.C.M., P.H. and A.A., and in collaboration with F.C., C.H.B., S.B., K.K.G., A.V., B.J.B., and C.A. All authors read and approved the final version of the article.

## Data sharing statement

Data are available upon reasonable request to the RODAM study coordinator (dr. Erik Beune, e.j.beune@amsterdamumc.nl).

## Declaration of interests

E.L.L. is a voluntary member of the junior council of Amsterdam Public Health Research Institute, Global Health section. All other authors declared no conflicts.
