# Supplementary material for: Epigenome-wide association study of plasma lipids in West Africans: the RODAM study
Source: eBioMedicine. 2023 Feb 13;89:104469. doi: 10.1016/j.ebiom.2023.104469 (PMC10025759; doi:10.1016/j.ebiom.2023.104469)

Table of Contents

Supplementary Table 1 – Study characteristics of independent cohorts from South African Batswana, African Americans and European cohorts
Supplementary Table 2 – Population characteristics, stratified by sex
Supplementary Table 3 – Top differentially methylated positions from independent cohorts from South African Batswana, African American and European cohorts replicated in RODAM study (candidate-gene approach)
Supplementary Table 4 – Summary statistics for top differentially methylated positions associated with lipid traits, after exclusion of participants with diabetes mellitus
Supplementary Table 5 – Comparison of DNA methylation levels and expression level correlation with the iMETHYL database and EWAS Toolkit
Supplementary Figure 1 – Participants inclusion flow chart
Supplementary Figure 2 – Principal components analysis for demographic variates (a), technical variates (b) and blood cell distribution (c)
Supplementary Figure 3 – QQ plots of the EWAS of lipids in the total population and of the meta-analysis of EWAS of lipids per geographical location
Supplementary Figure 4 – Manhattan plots for TC (a), LDL-C (b), HDL-C (c), triglycerides (d) of the meta-analysis of the EWAS per geographical location

Supplementary Table 1 – Study characteristics of independent cohorts from South African Batswana, African Americans and European cohorts

**Population characteristics**	**PURE-SA-NW (South African Bataswana)**	**Meta-analysis African descent (African American)**	**Meta-analysis European descent (European)**
N	120	4,452	11,114
Ethnicity	Batswana	African American	European descent
Sex, male (%)	100	24	40
Age (years)	64 [55-70]	58±6.9	61±7.9
BMI (kg/m2)	22.5 ±4.9	31.2 ±6.8	28.0±5.1
TC (mmol/L)	4.42 ±1.08	NA	NA
LDL-C (mmol/L)	2.50 ±0.92	3.38 ±0.99	3.31 ±0.91
HDL-C (mmol/L)	1.40 ±0.59	1.43 ±0.41	1.41 ±0.38
Triglycerides (mmol/L)	0.55 ±0.34	1.33 ±0.73	1.46 ±0.78
**DNA methylation analysis**			
Sample	Peripheral blood leukocytes isolated from whole blood	Peripheral blood leukocytes isolated from whole blood	Peripheral blood leukocytes isolated from whole blood
Array	Illumina Infinium MethylationEPIC BeadChip	Illumina Infinium HumanMethylation450 BeadChip	Illumina® Infinium HumanMethylation450 BeadChip
Transformation lipid	TC, LDL-C, HDL-C: no transformation<br>Triglycerides: log-transformed	TC, LDL-C: no transformation<br>HDL-C, triglycerides: natural log-transformed	
Analysis	TC, LDL-C, HDL-C:<br>DNAm ~ TC/LDL-C/HDL-C + age + WBC + SurrogateVars<br><br>Triglycerides:<br>DNAm ~ TG + age + BMI + WBC + SurrogateVars	Linear mixed models per cohort:<br>DNAm ~ lipid + age + sex + smoking + top four principal components from genotypes + WBC + plate, row, column<br><br>excl. those on lipid-lowering medication<br><br>Random effects meta-analysis with genomic control and Bonferonni correction (P<1.09*10^-7)	Linear mixed models per cohort:<br>DNAm ~ lipid + age + sex + smoking + top four principal components from genotypes + WBC + plate, row, column<br><br>excl. those on lipid-lowering medication<br><br>Random effects meta-analysis with genomic control and Bonferonni correction (P<1.09*10^-7)

Mean±standard deviation, median [interquartile range], HDL-C, high-density lipoprotein cholesterol; LDL-C, low-density lipoprotein cholesterol; TC, total cholesterol, TG, triglycerides; BMI, body mass index; WBC, white blood cell count

Supplementary Table 2 - Population characteristics, stratified by sex

	Total			Females						Males					
	Female	Male	p-value	Rural Ghana	Urban Ghana	Amsterdam	Berlin	London	p-value	Rural Ghana	Urban Ghana	Amsterdam	Berlin	London	p-value
n	382	281		69	168	56	23	66		32	71	83	52	43	
Age (mean (SD))	50.02 (10.42)	51.55 (9.25)	0.05	56.55 (9.11)	48.75 (9.75)	47.32 (8.93)	43.70 (9.55)	50.89 (11.68)	<0.001	55.47 (8.39)	54.89 (8.43)	49.82 (7.19)	48.00 (11.11)	50.77 (9.84)	<0.001
BMI (mean (SD))	27.56 (6.02)	25.61 (4.44)	<0.001	23.53 (4.16)	27.05 (6.03)	30.48 (5.40)	28.28 (4.93)	30.34 (5.88)	<0.001	21.27 (4.40)	24.08 (4.13)	26.67 (3.49)	26.86 (4.02)	27.81 (4.24)	<0.001
Diabetes mellitus (%)	123 (32.2)	108 (38.4)	0.114	24 (34.8)	55 (32.7)	16 (28.6)	7 (30.4)	21 (31.8)	0.962	16 (50.0)	30 (42.3)	29 (34.9)	21 (40.4)	12 (27.9)	0.311
Alcohol intake (units/day) (median [IQR])	0.00 [0.00, 0.03]	0.00 [0.00, 0.23]	<0.001	0.00 [0.00, 0.07]	0.00 [0.00, 0.00]	0.00 [0.00, 0.07]	0.03 [0.00, 0.18]	0.00 [0.00, 0.00]	0.001	0.03 [0.00, 0.27]	0.00 [0.00, 0.08]	0.00 [0.00, 0.17]	0.28 [0.06, 1.53]	0.00 [0.00, 0.00]	<0.001
Smoking (%)			<0.001						0.617						0.001
No, but I used to smoke	10 (2.7)	51 (18.8)		1 (1.5)	4 (2.4)	3 (5.6)	1 (4.3)	1 (1.59)		10 (34.5)	18 (25.7)	9 (11.1)	10 (19.6)	4 (9.8)	
No, I have never smoked	362 (97.3)	207 (76.1)		67 (98.5)	160 (97.6)	51 (94.4)	22 (95.7)	62 (98.4)		19 (65.5)	51 (72.9)	69 (85.2)	33 (64.7)	35 (85.4)	
Yes	0 (0.0)	14 (5.1)		0 (0.0)	0 (0.0)	0 (0.0)	0 (0.0)	0 (0.0)		0 (0.0)	1 (1.4)	3 (3.7)	8 (15.7)	2 (4.9)	
Length of Stay in Europe (years) (mean (SD))	18.84 (10.70)	18.31 (8.86)	0.639	NaN (NA)	NaN (NA)	19.38 (7.62)	18.74 (10.44)	18.39 (13.13)	0.889	NaN (NA)	NaN (NA)	18.75 (7.54)	19.20 (10.46)	16.28 (8.97)	0.254
Blood cell distribution (%) (mean (SD))															
CD8$^+$ T lymphocytes	0.12 (0.04)	0.10 (0.05)	<0.001	0.13 (0.04)	0.12 (0.04)	0.11 (0.03)	0.11 (0.04)	0.10 (0.04)	<0.001	0.11 (0.06)	0.10 (0.05)	0.10 (0.06)	0.09 (0.05)	0.09 (0.04)	0.322
CD4$^+$T	0.18 (0.06)	0.18 (0.06)	0.134	0.18 (0.05)	0.19 (0.06)	0.19 (0.05)	0.18 (0.06)	0.18 (0.06)	0.981	0.17 (0.06)	0.17 (0.06)	0.18 (0.06)	0.18 (0.06)	0.19 (0.05)	0.351
NK cells	0.10 (0.05)	0.12 (0.06)	<0.001	0.12 (0.06)	0.10 (0.05)	0.07 (0.04)	0.08 (0.04)	0.08 (0.04)	<0.001	0.15 (0.06)	0.14 (0.07)	0.10 (0.05)	0.12 (0.05)	0.13 (0.05)	<0.001
B cells	0.11 (0.03)	0.10 (0.04)	0.017	0.12 (0.04)	0.11 (0.03)	0.10 (0.03)	0.10 (0.03)	0.10 (0.03)	0.019	0.10 (0.04)	0.11 (0.04)	0.10 (0.04)	0.10 (0.03)	0.10 (0.03)	0.264
Monocytes	0.08 (0.02)	0.08 (0.02)	0.773	0.08 (0.02)	0.08 (0.03)	0.08 (0.02)	0.08 (0.02)	0.08 (0.03)	0.321	0.08 (0.03)	0.08 (0.03)	0.07 (0.02)	0.09 (0.03)	0.08 (0.02)	0.096
Granulocytes	0.45 (0.09)	0.45 (0.10)	0.666	0.41 (0.09)	0.44 (0.08)	0.49 (0.08)	0.48 (0.09)	0.48 (0.09)	<0.001	0.43 (0.12)	0.43 (0.10)	0.47 (0.09)	0.46 (0.10)	0.46 (0.08)	0.049
Lipid profile (mmol/L) (median[IQR])															
TC	5.24 [4.51, 6.08]	4.98 [4.35, 5.76]	0.084	4.93 [4.06, 5.73]	5.54 [4.73, 6.27]	5.28 [4.75, 5.91]	4.76 [3.79, 5.27]	4.98 [4.54, 5.56]	<0.001	4.31 [3.74, 5.04]	5.20 [4.36, 5.94]	5.08 [4.31, 5.73]	5.38 [4.69, 6.14]	4.98 [4.56, 5.72]	0.003
LDL-C	3.34 [2.73, 3.98]	3.20 [2.66, 3.92]	0.356	3.04 [2.36, 3.85]	3.62 [2.93, 4.23]	3.34 [2.79, 3.81]	2.82 [2.08, 3.28]	3.04 [2.77, 3.76]	<0.001	2.66 [2.29, 3.05]	3.33 [2.84, 3.97]	3.20 [2.62, 3.96]	3.34 [2.85, 3.92]	3.44 [2.91, 3.99]	0.003
HDL-C	1.34 [1.15, 1.56]	1.22 [1.05, 1.45]	<0.001	1.20 [1.05, 1.36]	1.30 [1.14, 1.52]	1.45 [1.14, 1.77]	1.47 [1.25, 1.71]	1.45 [1.30, 1.63]	<0.001	1.14 [0.89, 1.27]	1.15 [0.98, 1.35]	1.26 [1.06, 1.46]	1.39 [1.22, 1.62]	1.16 [1.10, 1.33]	<0.001
Triglycerides	0.97 [0.71, 1.40]	0.98 [0.74, 1.36]	0.686	1.10 [0.84, 1.62]	1.12 [0.83, 1.54]	0.76 [0.61, 1.06]	0.67 [0.50, 1.18]	0.79 [0.57, 1.07]	<0.001	1.08 [0.80, 1.38]	1.05 [0.82, 1.43]	0.90 [0.69, 1.28]	0.96 [0.77, 1.48]	0.96 [0.68, 1.21]	0.094

** p-values represent the comparison between the geographical locations, using one-way ANOVA to compare normally distributed continuous variables, Kruskal-Wallis test for non-normally distributed continuous variables, and Chi-square test for categorical variables.*

HDL-C, high-density lipoprotein cholesterol; LDL-C, low-density lipoprotein cholesterol; TC, total cholesterol; SD, standard deviation; IQR, interquartile range

Supplementary Table 3 - Top differentially methylated positions from independent cohorts from South African Batswana, African American and European cohorts replicated in RODAM study (candidate-gene approach)

In bold the transferred differentially methylated positions that were significantly associated after adjustment for multiple testing

** Annotated using UCSC catalogue*

*** RODAM model: DNAm ~ Lipid + sex + age + estimated cell proportions + batch + plate position + BMI + diabetes (transformation: HDL, LDL and TC inverse normalised transformed; triglycerides were natural log-transformed)*

*** *Direction of effect in each of the five sites, represented in order Amsterdam-Berlin-London-Rural Ghana-Urban Ghana; negative sign means negative direction of effect, positive sign means positive direction of effect*

**South African Batswana**						
**LDL-C (0/2) - South African Batswana**						
CpG	chr	pos	Gene symbol*	Regression Coefficient B**	Direction of effect***	p-value
cg15819853	chr8	8750517	*MFHAS1*	-0.005	+----	0.7116
cg01142936	chr17	79604293	*NPLOC4*	0	+---+	0.998
**HDL (0/16) - South African Batswana**						
CpG	chr	pos	Gene symbol*	Regression Coefficient B**	Direction of effect***	p-value
cg01042246	chr2	171572902	*SP5*	-0.0649	-+-+-	0.01193
cg23636606	chr14	95236539	*GSC*	-0.0336	+-++-	0.08722
cg24218109	chr6	80341034	*SH3BGRL2;SH3BGRL2*	-0.0341	-++--	0.1704
cg23256150	chr12	100867758	*NR1H4;NR1H4*	0.02	+-+++	0.2054
cg04139981	chr3	186982969	*MASP1;MASP1;MASP1*	0.0138	++-++	0.234
cg09775312	chr10	93392839	*PPP1R3C;PPP1R3C*	-0.025	+--+-	0.2989
cg15034009	chr15	26425086	intergenic	0.0137	+--++	0.4572
cg25830548	chr6	87041914	intergenic	0.0074	+--++	0.4887
cg13499300	chr19	54369556	*MYADM*	-0.015	-+-+-	0.5271
cg20585358	chr2	98354223	*ZAP70;ZAP70*	0.0079	+---+	0.5438
cg16168406	chr11	72414453	*ARAP1;ARAP1;ARAP1*	0.0093	++--+	0.5519
cg15114672	chr5	82766884	*VCAN;VCAN;VCAN;VCAN*	-0.0104	-++--	0.5606
cg04696808	chr16	34257170	intergenic	0.0062	--+++	0.6712
cg02801359	chr20	58508922	intergenic	-0.0056	-+++-	0.7913
cg15445037	chr20	60348161	*CDH4*	-0.0046	-+--+	0.8257
cg04608330	chr12	45269318	*NELL2;NELL2;NELL2;NELL2;NELL2*	0	-++++	0.9987
**Triglycerides (0/9) - South African Batswana**						
CpG	chr	pos	Gene symbol*	Regression Coefficient B**	Direction of effect***	p-value
cg23507945	chr6	137477184	*IL22RA2;IL22RA2;IL22RA2*	0.0259	++-+-	0.3551
cg26945643	chr7	2695248	*TTYH3*	-0.0214	----+	0.4549
cg12985237	chr5	6739530	*POLS*	0.0184	+++-+	0.4674
cg16149053	chr10	15413461	*FAM171A1*	0.0199	---++	0.5442
cg16806794	chr7	152591581	intergenic	0.0092	+-+++	0.7215
cg19222480	chr20	44441446	*UBE2C;UBE2C;UBE2C;UBE2C;UBE2C;U*	-0.0076	--+-+	0.8557
cg00925778	chr10	105113085	intergenic	0.0039	-+--+	0.8894
cg12913587	chr5	1232923	*SLC6A18*	-0.007	---++	0.8906
cg11582226	chr13	77587296	*FBXL3*	-0.0025	---++	0.9421
**African Americans**						
**LDL-C (3/5) p<0.01 - African Americans**						
CpG	chr	pos	Gene symbol*	Regression Coefficient B**	Direction of effect***	p-value
**cg00959259**	**chr3**	**122281975**	***PARP9***	**0.0703**	**-++++**	**0.001401**
**cg08122652**	**chr3**	**122281939**	***PARP9***	**0.0552**	**+-+++**	**0.001412**
**cg22930808**	**chr3**	**122281881**	***PARP9***	**0.0605**	**+++++**	**0.003666**
cg06188083	chr10	91093005	*IFIT3*	0.0291	-++++	0.086
cg16000331	chr22	42230138	*SREBF2*	0.0028	---++	0.867
**HDL-C (1/7) p<0.007 - African Americans**						
CpG	chr	pos	Gene symbol*	Regression Coefficient B**	Direction of effect***	p-value
**cg10177197**	**chr1**	**55316481**	***DHCR24***	**-0.0358**	**---+-**	**0.005905**
cg17901584	chr1	55353706	*DHCR24*	0.034	+++-+	0.06263
cg06500161	chr21	43656587	*ABCG1;ABCG1;ABCG1;ABCG1;ABCG1;A*	-0.0131	----+	0.2175
cg00607627	chr16	28995994	*LAT;LAT;LAT;LAT*	-0.0146	-+++-	0.2507
cg24002003	chr15	101668143	intergenic	0.0097	-+++-	0.4043
cg04682977	chr7	1062711	*C7orf50;MIR339;C7orf50;C7orf50*	0.0085	+-+-+	0.4379
cg02650017	chr17	47301614	*PHOSPHO1;PHOSPHO1*	-0.0024	-+++-	0.8962
**Triglycerides (4/43) p<0.001 - African Americans**						
CpG	chr	pos	Gene symbol*	Regression Coefficient B**	Direction of effect***	p-value
**cg19693031**	**chr1**	**145441552**	***TXNIP***	**-0.2637**	**-----**	**1.48E-09**
**cg17058475**	**chr11**	**68607737**	***CPT1A***	**-0.225**	**-----**	**1.93E-06**
**cg06500161**	**chr21**	**43656587**	***ABCG1***	**0.0993**	**+++++**	**1.51E-05**
**cg00574958**	**chr11**	**68607622**	***CPT1A***	**-0.1797**	**-----**	**0.0001489**
cg09737197	chr11	68607675	*CPT1A*	-0.1341	-----	0.00359
cg14476101	chr1	120255992	*PHGDH*	-0.1211	-----	0.006697
cg18336453	chr6	43082296	*PTK7*	-0.0531	-----	0.008977
cg08857797	chr17	40927699	*VPS25*	0.0736	+++++	0.01448
cg12001357	chr2	233410852	*CHRNG*	-0.1021	-----	0.01583
cg19266329	chr1	145456128	intergenic	-0.0761	-----	0.02052
cg16246545	chr1	120255941	*PHGDH*	-0.0724	-----	0.0211
cg20544516	chr17	17717183	*MIR33B*	0.0562	+++++	0.02411
cg11376147	chr11	57261198	*SLC43A1*	-0.0511	---+-	0.02895
cg19213703	chr3	177554561	intergenic	-0.0561	--+-+	0.06835
cg07504977	chr10	102131012	intergenic	0.061	+++++	0.09173
cg11024682	chr17	17730094	*SREBF1*	0.0377	+--++	0.09203
cg26403843	chr5	158634085	*RNF145*	0.0702	+++-+	0.0952
cg01881899	chr21	43652704	*ABCG1*	0.0694	+++-+	0.1012
cg19695041	chr8	38615330	*TACC1*	-0.0266	---+-	0.1335
cg16505233	chr3	5239311	*EDEM1*	-0.046	--++-	0.1348
cg10919522	chr14	74227441	*C14orf43*	-0.0592	----+	0.1363
cg02370100	chr21	43655256	*ABCG1*	-0.0347	--++-	0.1542
cg06690548	chr4	139162808	*SLC7A11*	-0.0512	---++	0.1743
cg09935388	chr1	92947588	*GFI1*	-0.0578	--+-+	0.1853
cg03725309	chr1	109757585	*SARS*	-0.0439	---++	0.1859
cg00008629	chr9	115093661	*ROD1*	-0.0767	-++--	0.188
cg05603985	chr1	2161049	*SKI*	-0.0301	---+-	0.2151
cg12593793	chr1	156074135	intergenic	-0.0289	+----	0.2401
cg12417689	chr17	259426	*C17orf97*	-0.0268	----+	0.2889
cg18696027	chr15	45002597	*B2M*	-0.0452	++-+-	0.3046
cg07661704	chr4	139144433	*SLC7A11*	-0.041	-----	0.3076
cg01979157	chr1	2161013	*SKI*	-0.054	--++-	0.3661
cg08884752	chr1	2162001	*SKI*	-0.0186	----+	0.4424
cg22304262	chr19	47287778	*SLC1A5*	-0.0245	-----	0.4425
cg03717755	chr6	16136539	*MYLIP*	0.0263	-+-++	0.4451
cg15863539	chr17	17716950	*SREBF1*	0.0181	+++++	0.454
cg27241845	chr2	233250370	intergenic	-0.0195	----+	0.5497
cg14170545	chr9	107688675	*ABCA1*	0.0288	++-++	0.5515
cg08309687	chr21	35320596	intergenic	-0.019	+-++-	0.579
cg10589813	chr20	48809978	intergenic	0.0087	---++	0.7184
cg11080651	chr5	10445523	*ROPN1L*	0.0071	+-++-	0.7521

CpG	chr	pos	Gene symbol*	Regression Coefficient B**	Direction of effect***	p-value
cg07397296	chr21	43655316	*ABCG1*	-0.0063	--+-+	0.842
cg09469355	chr1	2161886	*SKI*	-0.0018	+---+	0.9384
**Europeans**						
**LDL-C (0/15) p<0.003 - Europeans**						
CpG	chr	pos	Gene symbol*	Regression Coefficient B**	Direction of effect***	p-value
cg22930808	chr3	122281881	*PARP9;PARP9;DTX3L;PARP9;PARP9;PA*	0.0604	+++++	0.004536
cg16306978	chr2	21266953	*APOB*	0.0821	+-+++	0.008033
cg07839457	chr16	57023022	*NLRC5*	0.073	-++++	0.01304
cg25071744	chr2	21266995	*APOB*	0.0413	--+++	0.01698
cg00673290	chr2	21266727	*APOB*	0.0848	+-+++	0.01781
cg16723488	chr2	21266947	*APOB*	0.0574	--+++	0.01901
cg24309555	chr2	21266956	*APOB*	0.0393	--+++	0.027
cg05337441	chr2	21266568	*APOB*	0.0683	--+++	0.03548
cg03350299	chr2	21266960	*APOB*	0.0391	--+++	0.03938
cg21645268	chr8	11663584	*FDFT1*	0.0356	-++++	0.05464
cg11126134	chr13	31480304	*C13orf33*	0.053	+++++	0.05513
cg25536676	chr1	55353327	*DHCR24*	0.0266	-++++	0.08239
cg05119988	chr4	166251189	*SC4MOL;SC4MOL*	0.0229	--+++	0.1651
cg09984392	chr8	126011784	*SQLE*	0.028	-+-++	0.265
cg00285394	chr8	126011954	*SQLE*	0.0139	+--++	0.442
**HDL-C (1/69) p<0.0007 - Europeans**						
CpG	chr	pos	Gene symbol*	Regression Coefficient B**	Direction of effect***	p-value
**cg07839457**	**chr16**	**57023022**	***NLRC5***	**0.111**	**-++++**	**0.0003089**
cg08159663	chr16	57022486	*NLRC5*	0.0348	-++++	0.000964
cg17260706	chr11	118782879	*BCL9L*	0.028	+++++	0.002647
cg26804423	chr7	8201134	*ICA1;ICA1;ICA1*	-0.0314	----+	0.0165
cg06559575	chr12	53490352	*IGFBP6*	0.0257	+++++	0.02299
cg15609237	chr2	179395885	*TTN;TTN;TTN;MIR548N;TTN*	0.0275	+-+-+	0.0348
cg03676485	chr7	2563816	*LFNG;LFNG;LFNG;LFNG*	-0.0315	-----	0.03931
cg06560379	chr6	44231305	*NFKBIE*	0.0316	-++++	0.06141
cg26955383	chr10	105218660	*CALHM1*	-0.0203	----+	0.06429
cg17901584	chr1	55353706	*DHCR24*	0.0341	+++-+	0.06459
cg05119988	chr4	166251189	*SC4MOL;SC4MOL*	0.0296	--+++	0.07774
cg17194270	chr22	39759992	*SYNGR1;SYNGR1;SYNGR1*	-0.0599	---+-	0.1039
cg10639435	chr8	146104221	*ZNF250;ZNF250*	0.0195	+--++	0.1101
cg07175797	chr16	50317656	intergenic	-0.0124	-----	0.1198
cg07567724	chr1	153777721	*GATAD2B*	-0.024	-+--+	0.1353
cg20954977	chr2	232260116	*B3GNT7*	-0.0221	-+-+-	0.1411
cg07814318	chr15	31624584	*KLF13*	-0.0206	----+	0.1505
cg09259843	chr2	25452362	intergenic	0.0114	-+--+	0.1758
cg04934530	chr22	47080171	intergenic	-0.0388	-----	0.1782
cg06500161	chr21	43656587	*ABCG1;ABCG1;ABCG1;ABCG1;ABCG1;A*	-0.0134	----+	0.1986
cg01003245	chr1	151428287	*POGZ;POGZ;POGZ*	-0.0206	----+	0.2014
cg00567854	chr1	203273693	*BTG2*	-0.0113	-+---	0.2029
cg19273683	chr1	21656047	*ECE1*	-0.0123	----+	0.2382
cg06192883	chr15	52554171	*MYO5C*	-0.015	----+	0.2636
cg01101459	chr1	234871477	intergenic	-0.0198	++--+	0.2684
cg05008975	chr11	118022317	*SCN4B;SCN4B*	-0.0087	----+	0.2923
cg11024682	chr17	17730094	*SREBF1;SREBF1*	-0.0104	--++-	0.3
cg15804598	chr17	43224418	*HEXIM1*	0.0089	--+-+	0.3127
cg15636859	chr20	55982844	*RBM38;RBM38*	0.0165	+-+++	0.3138
cg15706973	chr2	111633044	*ACOXL;ACOXL;ACOXL*	0.0151	++--+	0.3439
cg03819286	chr16	4673974	*MGRN1;MGRN1;MGRN1;MGRN1*	0.0118	+--++	0.3572
cg13222915	chr1	184598594	intergenic	0.0079	++-++	0.3696
cg24002003	chr15	101668143	intergenic	0.01	-+++-	0.385
cg02578470	chr3	127320670	*MCM2*	-0.0103	++---	0.3905
cg10438589	chr4	14531493	intergenic	-0.009	---++	0.4296
cg09199486	chr17	48259448	intergenic	-0.0053	+----	0.4318
cg09039672	chr12	52367989	*ACVR1B;ACVR1B;ACVR1B*	-0.007	+---+	0.445
cg15551881	chr9	123688715	*TRAF1*	-0.0119	+---+	0.4644
cg21486834	chr17	74477542	*RHBDF2;RHBDF2*	0.0104	+-+-+	0.4891
cg02855207	chr11	73087016	*RELT;RELT*	-0.0062	+---+	0.5141
cg17485681	chr10	73565625	*CDH23*	0.0095	++-+-	0.5218
cg19351166	chr2	209133632	*PIKFYVE;PIKFYVE*	0.0105	++--+	0.5276
cg22312249	chr17	72779428	*TMEM104*	0.0095	+---+	0.562
cg12458003	chr1	204960826	*NFASC;NFASC;NFASC;NFASC*	0.0064	----+	0.5955
cg16407699	chr10	74020428	intergenic	-0.0067	+----	0.6033
cg04577162	chr7	73667397	*RFC2;RFC2*	-0.0063	---++	0.6036
cg02879453	chr16	50321818	*ADCY7*	0.0059	++--+	0.6556
cg01944226	chr17	80188056	*SLC16A3;SLC16A3*	0.0073	+--++	0.669
cg25551219	chr22	50909865	*SBF1*	-0.0038	----+	0.6784
cg24843003	chr19	1409547	*DAZAP1;DAZAP1*	-0.0037	--+-+	0.6959
cg10073091	chr1	55352301	*DHCR24*	0.0058	-+++-	0.6966
cg12825509	chr3	185648568	*TRA2B*	-0.0059	---++	0.6995
cg25921813	chr3	194797138	*C3orf21*	0.0101	---++	0.7302
cg11969813	chr17	79816559	*P4HB*	0.0069	+---+	0.7327
cg23580000	chr16	50322156	*ADCY7*	-0.0036	----+	0.746
cg06897661	chr16	50322074	*ADCY7;ADCY7*	-0.003	----+	0.7957
cg24678869	chr1	153919638	*DENND4B*	-0.0017	-+--+	0.8181
cg20784591	chr7	99972461	*PILRA;PILRA;PILRA*	-0.0016	-+-++	0.859
cg02650017	chr17	47301614	*PHOSPHO1;PHOSPHO1*	-0.0029	-+++-	0.8776
cg27243685	chr21	43642366	*ABCG1;ABCG1;ABCG1;ABCG1;ABCG1;A*	0.0023	+--++	0.8964
cg25739715	chr22	30663881	*OSM*	0.0017	-++++	0.9078
cg19881557	chr14	20967426	intergenic	-0.0016	++--+	0.9162
cg12194745	chr17	79423649	*BAHCC1*	-0.0011	-+--+	0.9177
cg22548088	chr19	6271786	*MLLT1*	0.0021	-++-+	0.9228
cg00732815	chr20	44636981	*MMP9*	0.0013	-+-++	0.9312
cg25217710	chr1	156609523	intergenic	-0.0013	++--+	0.9423
cg13821176	chr8	126448338	*TRIB1*	8.00E-04	+---+	0.956
cg14352682	chr6	30614168	*C6orf136;C6orf136;C6orf136*	-4.00E-04	----+	0.9612
cg05155595	chr2	70008161	*ANXA4*	-1.00E-04	+---+	0.9971
**Triglycerides (4/86) p<0.0006 - Europeans**						
CpG	chr	pos	Gene symbol*	Regression Coefficient B**	Direction of effect***	p-value
**cg19693031**	**chr1**	**145441552**	***TXNIP***	**-0.2636**	**-----**	**1.73E-09**
**cg17058475**	**chr11**	**68607737**	***CPT1A;CPT1A***	**-0.2248**	**-----**	**2.16E-06**
**cg06500161**	**chr21**	**43656587**	***ABCG1;ABCG1;ABCG1;ABCG1;ABCG1;***	**0.1001**	**+++++**	**1.08E-05**
**cg00574958**	**chr11**	**68607622**	***CPT1A;CPT1A***	**-0.1795**	**-----**	**0.0001613**
cg11969813	chr17	79816559	*P4HB*	0.145	+++++	0.001182
cg19420720	chr17	79816504	*P4HB*	0.0799	+++++	0.001249
cg02571436	chr10	77468337	intergenic	-0.1276	-----	0.001833
cg09737197	chr11	68607675	*CPT1A;CPT1A*	-0.134	-----	0.003759

cg25217710	chr1	156609523	intergenic	0.1056	+++++	0.006189
cg14476101	chr1	120255992	*PHGDH*	-0.1211	-----	0.006932
cg18336453	chr6	43082296	*PTK7;PTK7;PTK7;PTK7*	-0.0536	-----	0.007732
cg01676795	chr7	75586348	*POR*	0.0947	+++++	0.01071
cg05778424	chr17	55169508	*AKAP1*	0.0519	-++++	0.01197
cg08857797	chr17	40927699	*VPS25*	0.0737	+++++	0.01429
cg00948664	chr3	48935316	*SLC25A20*	-0.1063	+----	0.01762
cg04460609	chr4	16532808	*LDB2;LDB2*	-0.0576	-----	0.01818
cg19266329	chr1	145456128	intergenic	-0.0764	-----	0.01991
cg16246545	chr1	120255941	*PHGDH*	-0.0724	-----	0.02108
cg18120259	chr6	43894639	*LOC100132354*	-0.0574	-----	0.02266
cg20544516	chr17	17717183	*MIR33B;SREBF1;SREBF1*	0.0561	+++++	0.0237
cg02316713	chr21	43619559	*ABCG1;ABCG1*	0.0909	+++++	0.0272
cg11376147	chr11	57261198	*SLC43A1*	-0.0512	---+-	0.02758
cg13123009	chr6	31681882	*LY6G6E;LY6G6D;LY6G6E*	0.0411	+++-+	0.03121
cg01787285	chr1	2162682	*SKI*	-0.0938	--+--	0.03177
cg21291385	chr21	35448215	*MRPS6;SLC5A3*	-0.0586	---+-	0.03643
cg01176028	chr21	43653234	*ABCG1;ABCG1;ABCG1;ABCG1;ABCG1;*	0.0561	+++++	0.04301
cg15564619	chr1	2163437	*SKI*	-0.0646	+----	0.0513
cg24174557	chr17	57903544	*TMEM49*	-0.0865	---+-	0.05227
cg21429551	chr7	30635762	*GARS*	-0.089	-----	0.05768
cg19213703	chr3	177554561	intergenic	-0.0563	--+-+	0.06742
cg13740985	chr9	80930413	*PSAT1;PSAT1*	0.0358	+--++	0.07062
cg20702913	chr2	10183115	*KLF11*	-0.0694	--+--	0.08016
cg22911054	chr11	68603379	*CPT1A;CPT1A*	0.0598	--+++	0.0816
cg12556569	chr11	116664039	*APOA5;APOA5*	0.5027	+++-+	0.08925
cg07504977	chr10	102131012	intergenic	0.0609	+++++	0.09265
cg26403843	chr5	158634085	*RNF145*	0.0703	+++-+	0.09595
cg11024682	chr17	17730094	*SREBF1;SREBF1*	0.0368	+--++	0.0969
cg14868222	chr6	31548340	*LTB;LTB*	-0.0364	+-+--	0.09786
cg27243685	chr21	43642366	*ABCG1;ABCG1;ABCG1;ABCG1;ABCG1;*	0.0625	++-++	0.09932
cg01881899	chr21	43652704	*ABCG1;ABCG1;ABCG1;ABCG1;ABCG1;*	0.0692	+++-+	0.1036
cg17901584	chr1	55353706	*DHCR24*	-0.0664	----+	0.1037
cg10919522	chr14	74227441	*C14orf43;C14orf43*	-0.0591	----+	0.1385
cg16097041	chr1	154965544	*FLAD1;LENEP;FLAD1*	0.0388	+-+-+	0.1512
cg02370100	chr21	43655256	*ABCG1;ABCG1;ABCG1;ABCG1;ABCG1;*	-0.0346	--++-	0.1531
cg23599026	chr10	102268546	*SEC31B*	0.0256	+-+++	0.1685
cg05119988	chr4	166251189	*SC4MOL;SC4MOL*	-0.051	-+--+	0.1703
cg10192877	chr21	43641690	*ABCG1;ABCG1;ABCG1;ABCG1;ABCG1;*	0.0298	--+++	0.1742
cg06690548	chr4	139162808	*SLC7A11*	-0.0512	---++	0.1753
cg03725309	chr1	109757585	*SARS*	-0.0439	---++	0.1861
cg08549335	chr7	30387954	*ZNRF2*	-0.0678	-----	0.1907
cg19588519	chr10	125817817	intergenic	0.0282	-++++	0.204
cg26262157	chr10	6214079	*PFKFB3*	-0.0352	+--+-	0.2131
cg05014727	chr10	6214016	*PFKFB3*	-0.0464	+----	0.2163
cg26989316	chr11	68607257	*CPT1A;CPT1A*	-0.0639	---+-	0.2205
cg14442061	chr17	17740468	*SREBF1;SREBF1*	-0.0441	-++--	0.2312
cg02641801	chr2	26213508	intergenic	0.021	++-++	0.2699
cg08640824	chr1	2162506	*SKI*	-0.0511	-+-+-	0.2786
cg25741837	chr2	73452813	*SMYD5*	0.0263	-++++	0.2961
cg17713673	chr12	46466053	intergenic	-0.0341	-+-+-	0.3546
cg14959425	chr7	20447707	*ITGB8*	-0.0331	-+--+	0.3589
cg01438090	chr11	30502936	*MPPED2;MPPED2*	-0.0508	----+	0.3993
cg00716257	chr14	75897417	*JDP2;JDP2;JDP2;JDP2*	-0.024	++--+	0.421
cg01082498	chr11	68608225	*CPT1A;CPT1A*	-0.0383	---+-	0.4361
cg22304262	chr19	47287778	*SLC1A5;SLC1A5;SLC1A5*	-0.0244	-----	0.4435
cg03717755	chr6	16136539	*MYLIP*	0.0264	-+-++	0.4439
cg15863539	chr17	17716950	*SREBF1;SREBF1*	0.0182	+++++	0.4489
cg19202384	chr17	79894511	*PYCR1;PYCR1*	-0.0189	+----	0.5111
cg11851174	chr17	17712609	*RAI1*	0.0178	+-+-+	0.5481
cg07730360	chr3	128845626	intergenic	0.0139	-+--+	0.5668
cg08309687	chr21	35320596	intergenic	-0.0191	+-++-	0.5766
cg01538969	chr6	30624636	*DHX16;DHX16*	0.011	+--++	0.6081
cg23184690	chr19	45429771	*APOC1P1;APOC1P1;APOC1P1*	0.0083	----+	0.6343
cg02711608	chr19	47287964	*SLC1A5;SLC1A5;SLC1A5;SLC1A5*	-0.0181	---++	0.6841
cg08129017	chr17	17728660	*SREBF1;SREBF1*	0.0099	+-++-	0.7258
cg08994060	chr10	6214026	*PFKFB3*	-0.0117	+--+-	0.728
cg09173378	chr1	196370245	*KCNT2*	-0.0091	--+-+	0.7415
cg10639435	chr8	146104221	*ZNF250;ZNF250*	-0.008	-++--	0.7667
cg00222799	chr21	43655464	*ABCG1;ABCG1;ABCG1;ABCG1;ABCG1;*	-0.0076	--+++	0.7681
cg00042882	chr7	100796809	*AP1S1*	0.0102	++-++	0.7874
cg00177237	chr21	43658316	*ABCG1;ABCG1;ABCG1;ABCG1;ABCG1;*	0.0077	-+--+	0.8143
cg07397296	chr21	43655316	*ABCG1;ABCG1;ABCG1;ABCG1;ABCG1;*	-0.0062	--+-+	0.8463
cg03068497	chr7	30635838	*GARS*	-0.0076	-+--+	0.8501
cg14597545	chr15	73074210	*ADPGK;ADPGK;ADPGK*	0.0047	---++	0.876
cg06192883	chr15	52554171	*MYO5C*	0.0032	-+-++	0.9148
cg24694018	chr1	145457621	*POLR3GL*	-0.003	+++--	0.9221
cg19390658	chr7	30636176	*GARS*	0.0026	+---+	0.9382

Supplementary Table 4 – Summary statistics for top differentially methylated positions associated with lipid traits, after exclusion of participants with diabetes mellitus

				**Total population***			**Excluding diabetes mellitus****		
**TC**	chr	pos	Gene	Regres. Coef. Beta	Direction of effect***	p-value	Regres. Coef. Beta	Direction of effect***	p-value
cg19693031	chr1	145441552	*TXNIP*	-0.0957	-----	8.43E-07	-0.0612	-----	1.10E-02
cg03753191	chr13	43566902	*EPSTI1*	0.0997	+++++	3.54E-06	0.0905	-++++	1.40E-03
cg26816907	chr1	197890812	*LHX9*	0.0718	+++++	5.49E-06	0.0785	+++++	1.81E-04
cg11066601	chr1	185373486	intergenic	-0.2233	-+---	6.53E-06	-0.2327	-+-+-	2.81E-04
cg03167407	chr2	241261657	intergenic	0.1782	+++++	9.30E-06	0.1887	+++++	1.92E-04
**HDL**	chr	pos	Gene	Regres. Coef. Beta	Direction of effect***	p-value	Regres. Coef. Beta	Direction of effect***	p-value
cg05091570	chr1	201709336	*NAV1*	-0.0746	-----	9.09E-07	-0.0653	-+---	5.16E-04
cg07622193	chr19	42701920	intergenic	-0.0624	-----	1.68E-06	-0.0645	-+---	1.11E-04
cg00091964	chr2	80530891	*CTNNA2*	-0.0888	-----	2.19E-06	-0.0845	-+---	6.12E-04
cg13767294	chr17	41856619	*DUSP3*	-0.072	-----	5.23E-06	-0.0629	-+---	2.49E-03
cg08926253	chr11	614761	*IRF7*	0.0481	+++++	6.03E-06	0.046	+++++	1.29E-03
**LDL**	chr	pos	Gene	Regres. Coef. Beta	Direction of effect***	p-value	Regres. Coef. Beta	Direction of effect***	p-value
cg03753191	chr13	43566902	*EPSTI1*	0.0976	+++++	4.14E-06	0.0885	+++++	1.57E-03
cg26816907	chr1	197890812	*LHX9*	0.0679	+++++	1.36E-05	0.0807	+++++	8.50E-05
cg13781819	chr1	47469065	intergenic	-0.053	-----	3.40E-05	-0.0348	---+-	4.24E-02
cg20294940	chr14	105866596	intergenic	-0.049	-----	5.30E-05	-0.0277	-----	7.81E-02
cg23970275	chr2	208008052	*KLF7*	-0.0674	-----	5.75E-05	-0.0755	---+-	6.89E-04
**Triglycerides**	chr	pos	Gene	Regres. Coef. Beta	Direction of effect***	p-value	Regres. Coef. Beta	Direction of effect***	p-value
cg19693031	chr1	145441552	*TXNIP*	-0.2637	-----	1.67E-09	-0.2237	-----	5.43E-05
cg17058475	chr11	68607737	*CPT1A*	-0.225	-----	2.09E-06	-0.2018	-----	1.37E-03
cg06500161	chr21	43656587	*ABCG1*	0.1001	+++++	1.17E-05	0.1056	+++-+	8.36E-04
cg05697101	chr2	38829104	*HNRPLL*	-0.3446	-----	2.81E-05	-0.2351	-----	5.61E-02
cg11066601	chr1	185373486	intergenic	-0.4686	-----	3.34E-05	-0.4335	-----	4.54E-03

** Model: DNAm ~ Lipid + sex + age + estimated cell proportions + batch + plate position + BMI + diabetes (transformation: HDL-C , LDL-C and TC inverse normalised transformed; triglycerides were natural log-transformed)*

*** Model: DNAm ~ Lipid + sex + age + estimated cell proportions + batch + plate position + BMI (transformation: HDL-C , LDL-C and TC inverse normalised transformed; triglycerides were natural log-transformed)*

****Direction of effect in each of the five sites, represented in order Amsterdam-Berlin-London-Rural Ghana-Urban Ghana; negative sign means negative direction of effect, positive sign means positive direction of effect*

Supplementary Table 5 – Comparison of DNA methylation levels and expression level correlation with the iMETHYL database and EWAS Toolkit

CpG	Gene symbol	Gene feature	*RODAM - Methylation level, % (sd)**	*iMethyl - CD4+ T Methylation level***	*iMethyl - CD4+ T Methylation level, % (sd) **	*FPKM CD4+ T average (sd)*	*Subcutaneous adipose tissue-Methylation level, %****	*Visceral adipose tissue-Methylation level, %****	*Liver - Methylation level, %****	*Correlation *****	*p-value correlation*
TC											
cg19693031	*TXNIP*	3'UTR	78.52947 (6.361053)	High	81.4624 (11.9758)	3.18(0.13)	NA	NA	NA	-0.437	6.78E-20
cg03753191	*EPSTI1*	TSS1500	8.654768 (3.110533)	Low	2.21235 (4.50656)	NA	45.28	31.98	8.00	-0.503	1.08E-26
cg26816907	*LHX9*	Body	29.67202 (6.246431)	Low	33.3615 (8.92373)	NA	31.99	32.01	54.24	NA	NA
cg11066601	intergenic		78.68967 (11.31238)	High	76.1844 (18.5016)	NA	NA	NA	NA	NA	NA
cg03167407	intergenic		77.63261 (12.98674)	High	66.7636 (25.6234)	NA	NA	NA	NA	NA	NA
LDL-C											
cg03753191	*EPSTI1*	TSS1500	8.654768 (3.110533)	Low	2.21235(4.50656)	NA	45.28	31.98	8.00	0.539	3.39E-31
cg26816907	*LHX9*	Body	29.67202 (6.246431)	Low	33.3615 (8.92373)	NA	31.99	32.01	54.24	NA	NA
cg13781819	intergenic		88.94 (2.143102)	High	87.8024 (7.90864)	NA	NA	NA	NA	NA	NA
cg20294940	intergenic		92.37888 (1.609764)	High	97.4776 (2.25169)	-0.52 (0.20)	96.57	96.89	97.09	NA	NA
cg23970275	*KLF7*	Body	16.88181 (6.269963)	Low	9.80631 (10.0402)	1.29 (0.13)	NA	NA	NA	NA	NA
HDL-C											
cg05091570	*NAV1*	Body	2.827658 (0.82965)	Low	1.02845 (2.6817)	-0.05 (0.16)	32.18	26.50	10.26	0.308	4.09E-10
cg07622193	intergenic		11.82514 (3.330731)	Low	15.7993 (7.70943)	NA	40.01	32.10	39.52	NA	NA
cg00091964	*CTNNA2*	Body	3.906463 (1.459099)	Low	1.92697 (2.59557)	NA	1.25	1.68	2.08	NA	NA
cg13767294	*DUSP3*	TSS1500	4.474875 (1.13062)	Low	2.75212 (2.89408)	NA	3.26	3.13	3.26	NA	NA
cg08926253	*IRF7*	Body	56.44285 (4.352698)	High	77.9225 (6.49456)	1.45 (0.11)	46.54	45.53	33.30	-0.178	0.00037848
Triglycerides											
cg19693031	*TXNIP*	3'UTR	78.52947 (6.361053)	High	81.4624 (11.9758)	3.18(0.13)	NA	NA	NA	-0.437	6.78E-20
cg17058475	*CPT1A*	5'UTR	13.90628 (5.097375)	Low	21.3527 (10.6246)	1.09 (0.24)	1.92	8.85	4.94	-0.243	1.02E-06
cg06500161	*ABCG1*	Body	61.22299 (3.949012)	High	80.2526 (8.13768)	1.00 (0.12)	42.80	55.46	65.09	0.244	9.35E-07
cg05697101	*HNRPLL*	Body	8.238972 (3.754813)	Low	0.636651 (1.66883)	1.51 (0.15)	NA	NA	NA	NA	NA
cg11066601	intergenic		78.68967 (11.31238)	High	76.1844 (18.5016)	NA	NA	NA	NA	NA	NA

** calculated as beta value * 100*

*** low ≤33% methylated; medium = 34-66% methylated; high ≥67% methylated*

**** EWAS toolkit*

***** Pearson correlation between DNA methylation and expression in liver, EWAS toolkit*

HDL-C, high-density lipoprotein cholesterol; LDL-C, low-density lipoprotein cholesterol; TC, total cholesterol; SD, standard deviation; UTR, untranslated region; TSS, transcription start site; NA, not available

Supplementary Figure 1 – Participants inclusion flow chart

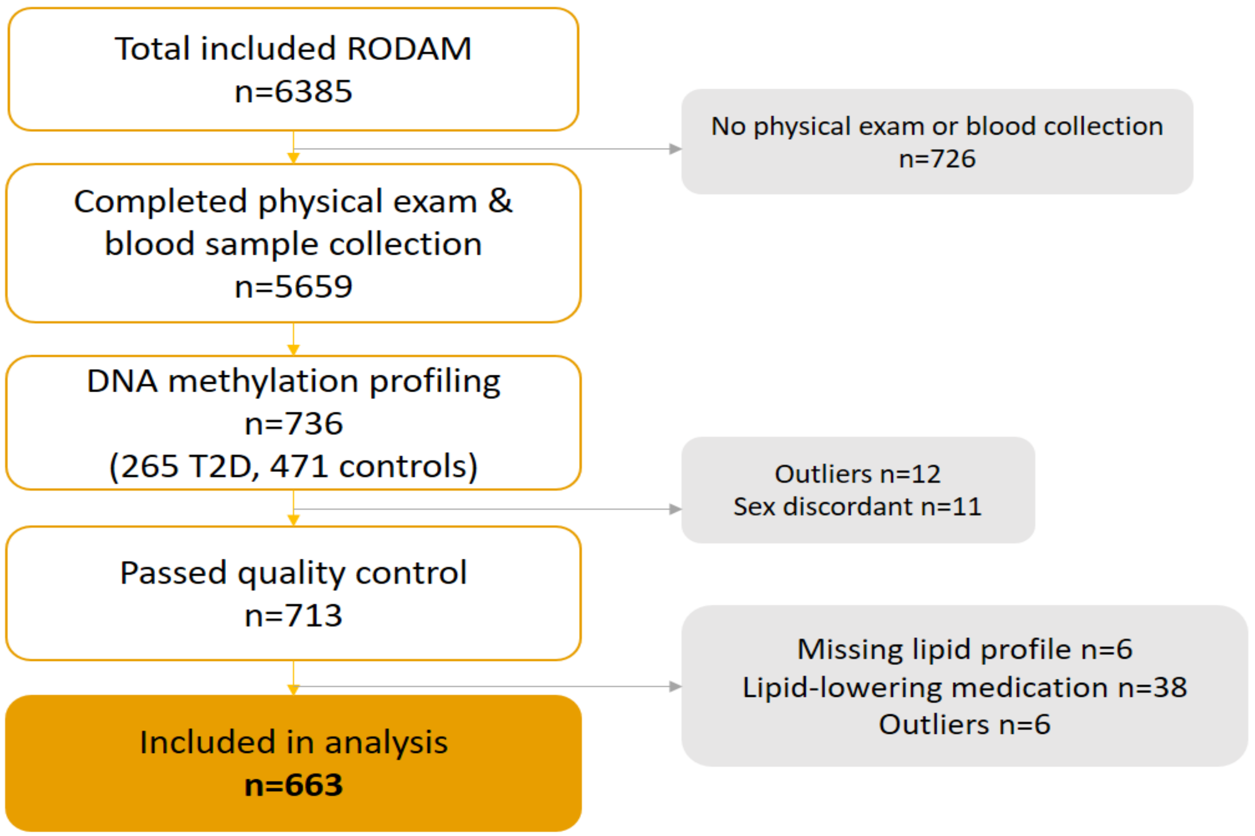

T2D, diabetes mellitus

Supplementary Figure 2 - Principal components analysis for demographic variates (a), technical variates (b) and blood cell distribution (c))

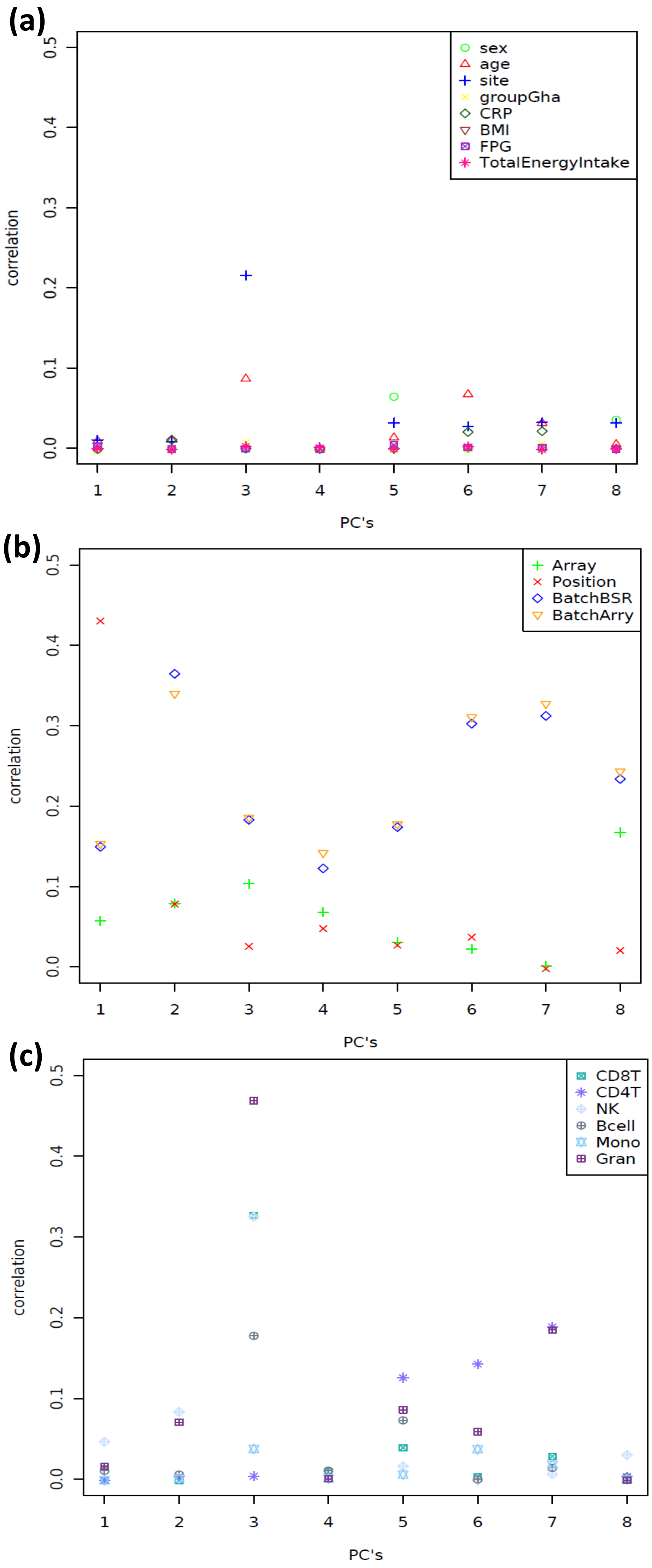

Supplementary Figure 3 – QQ plots of the meta-analysis of EWAS of lipids per geographical location

QQ plots for Total Cholesterol (a), LDL-C (b), HDL-C (c), triglycerides (d)

Model adjusted for age, sex, BMI, diabetes mellitus, estimated cell count, hybridisation batch and array position

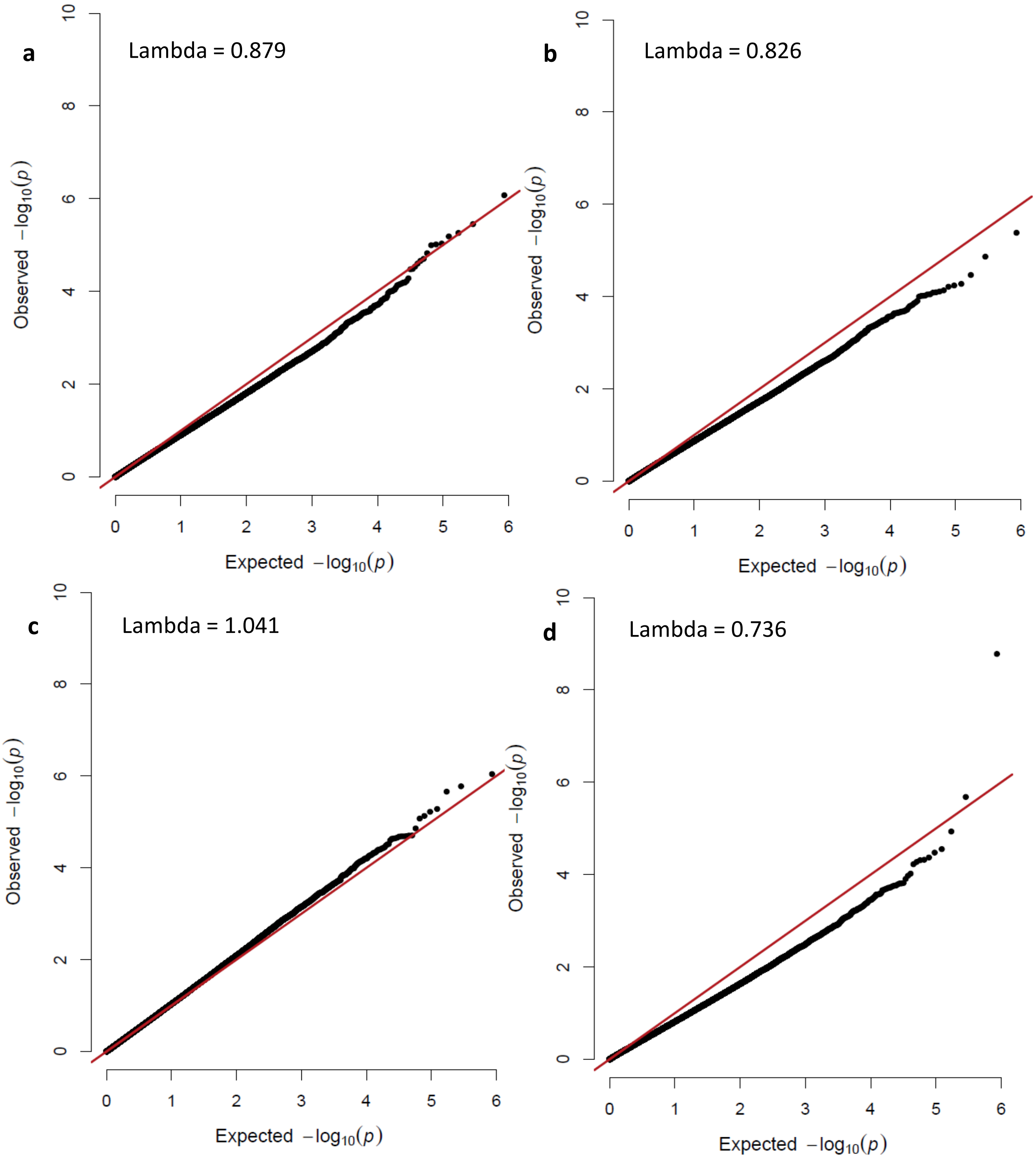

Supplementary Figure 4 - Manhattan plots for TC (a), LDL-C (b), HDL-C (c), triglycerides (d) of the meta-analysis of the EWAS per geographical location

Model adjusted for age, sex, BMI, diabetes mellitus, estimated cell count, hybridisation batch and array position

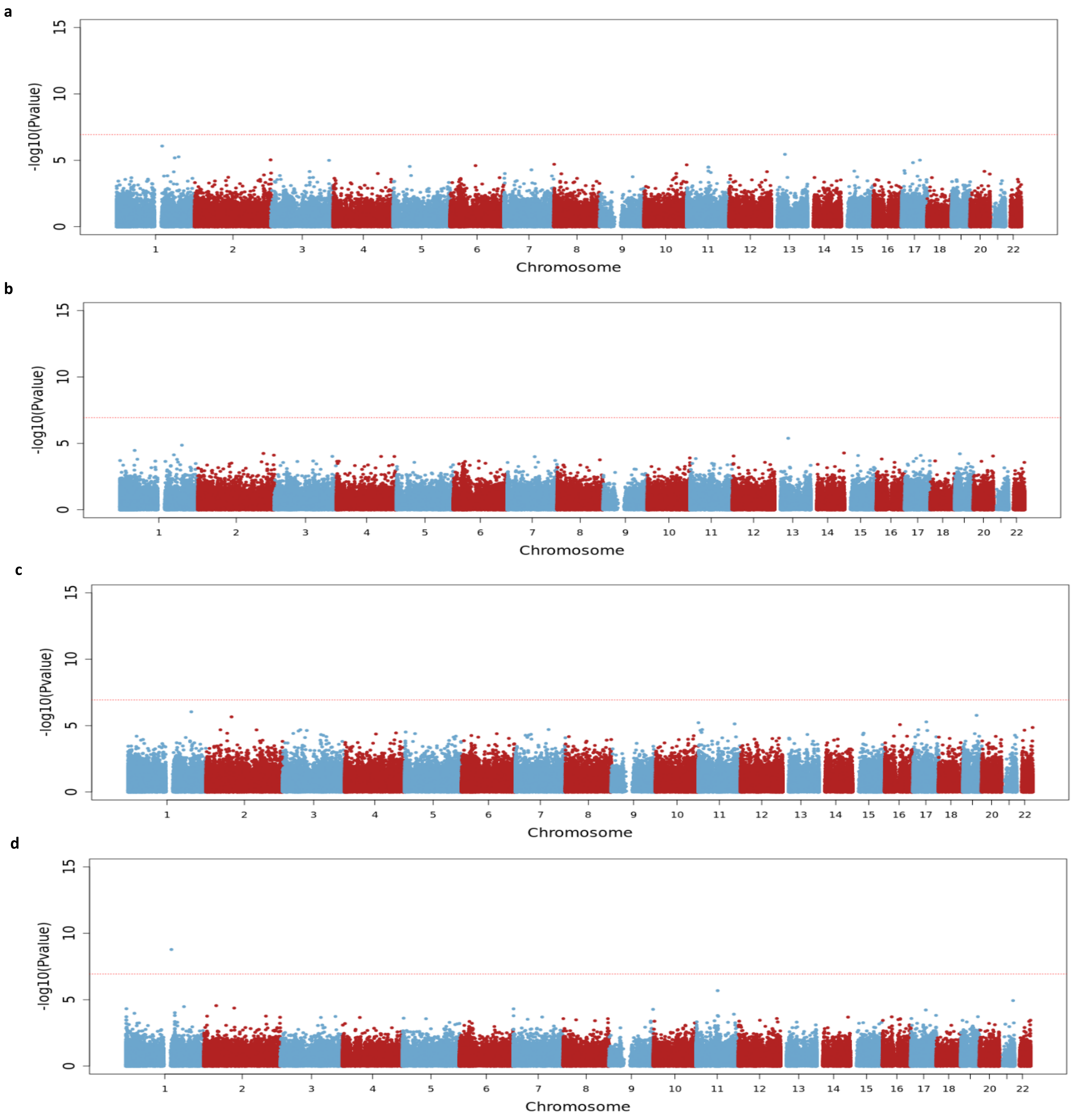

Supplement: Supplementary Figs. S1–S4 and Tables S1–S5 [file mmc1.pdf]
